# Polarization- and Angular-Resolved Optical Response of Molecules on Anisotropic Plasmonic Nanostructures

**DOI:** 10.3390/nano8060418

**Published:** 2018-06-09

**Authors:** Martin Šubr, Marek Procházka

**Affiliations:** Faculty of Mathematics and Physics, Institute of Physics, Charles University, 121 16 Prague 2, Czech Republic

**Keywords:** SERS, Raman, plasmonics, metallic nanostructures, polarization and angular dependences, molecular orientation, ellipsometry

## Abstract

A sometimes overlooked degree of freedom in the design of many spectroscopic (mainly Raman) experiments involve the choice of experimental geometry and polarization arrangement used. Although these aspects usually play a rather minor role, their neglect may result in a misinterpretation of the experimental results. It is well known that polarization- and/or angular- resolved spectroscopic experiments allow one to classify the symmetry of the vibrations involved or the molecular orientation with respect to a smooth surface. However, very low detection limits in surface-enhancing spectroscopic techniques are often accompanied by a complete or partial loss of this detailed information. In this review, we will try to elucidate the extent to which this approach can be generalized for molecules adsorbed on plasmonic nanostructures. We will provide a detailed summary of the state-of-the-art experimental findings for a range of plasmonic platforms used in the last ~ 15 years. Possible implications on the design of plasmon-based molecular sensors for maximum signal enhancement will also be discussed.

## 1. Introduction

Methods of optical spectroscopy, such as absorption spectroscopy, Raman scattering spectroscopy or fluorescence spectroscopy, can be performed in a wide range of experimental setups relying on a proper choice of instrumentation, light sources, waveguides, monochromators, detectors etc. Interplay between all the factors mentioned above determines the final spectral pattern observed in spectroscopic experiments. An obvious degree of freedom in the experimental design is the polarization and (especially in the case of Raman experiments) angular arrangement used. Although for many types of experiments this additional information is not required, variation in light polarization and/or the scattering angle give rise to a family of phenomena that both theoreticians and experimentalists should be aware of when interpreting their results. For example, confusion of laser polarization may lead to very strong Raman bands to appear as rather weak in the Raman spectra, and vice versa. Thus, understanding these effects helps to obtain deeper insight into the properties of the system studied.

In this review, the main emphasis is placed on polarization and directional/angular characteristics occurring in Raman spectroscopy and surface-enhanced Raman spectroscopy (SERS). However, it briefly touches on the polarization and angular dependences of other optical phenomena such as absorbance, reflectance or fluorescence. It is well known that polarization- and/or angular-resolved Raman experiments allow one to classify the symmetry of the vibrations involved [1,2,3] or molecular orientation with respect to a smooth surface [4,5,6]. We will try to extend some of these results to molecules adsorbed on plasmonic nanostructures. Generalizing these results to the case of SERS is not straightforward due to the existence of one more element—the nanostructure, which imposes its own anisotropic pattern in the polarization and directional properties of the SERS signal.

Although some reviews focusing on anisotropic metallic nanostructures for SERS and their fabrication already exist in literature [7,8,9,10], our review covers the topic from a rather untraditional point of view, focusing primarily on their polarization and directional properties. The review is organized as follows. Firstly, basic overview of polarization and angular dependences in classical Raman spectroscopy will be introduced. Different experimental geometries for Raman scattering measurements will be presented and consequences for the Raman signal coming from randomly oriented molecules as well as crystals will be derived. We will summarize basic results of the group theory, allowing classification of normal vibrational modes depending on the symmetry of the vibration involved. Theoretical predictions will be briefly compared to the experimental results measured in our laboratory. It is important to note that we will focus only on the phenomena within the dipole approximation, leaving aside the vast body of experimental techniques such as dichroic methods or Raman optical activity [11]. Secondly, we will concentrate on the polarization effects for molecules adsorbed on smooth surfaces. We will show that interference between the incident and the reflected radiation must be taken into account in order to correctly describe the observed spectral pattern. The crucial part will involve the derivation of the surface-selection rules, allowing determination of molecular orientation on smooth surfaces. Although this approach may seem to go far beyond the original scope of this review, we will show that a very similar principle also applies on roughened metallic surfaces and contributes to the SERS enhancement. Finally, we will try to generalize previous considerations to the case of surface-enhanced Raman scattering. We will summarize the up-to-date findings regarding polarization- and angular-resolved SERS experiments and survey the contributions to this field from the last 15 years. The influence of both different coupling efficiency of different laser polarizations to plasmons as well as symmetry of the Raman tensor of the analyte on polarized SERS will be critically discussed. Described phenomena will be supported by our own experimental results as well as by published ones.

## 2. The Origin of Polarization Characteristics in Spectroscopy

Before going deeply into the definitions of the depolarization ratios or formulations of the selection rules, we devote a short paragraph to the theoretical description of the polarization of the light waves. A monochromatic plane wave travelling along the z axis in a given Cartesian coordinate system can be characterized by its electric field vector in any instant restricted to the xy plane. Thus, the variables in an equation describing a plane wave can be decoupled in the form [12]:

(1) $$\vec{E}\left(x,y,z,t\right)=E_{0x}\cos\left(\omega t-kz\right){\vec{e}}_{x}+E_{0y}\cos\left(\omega t-kz+\delta \right){\vec{e}}_{y},$$

where $E_{0x}$ and $E_{0y}$ denote amplitudes of the electric field intensities in the x and y direction, respectively (traditionally, the polarization direction is assumed to be determined by the electric field vector instead of the magnetic field vector), k denotes the wavevector and ω the angular frequency. The way that the electric field vector oscillates in space and time defines the light polarization. In the simplest case, these oscillations are confined to a single spatial direction, which is termed linear polarization. In this case, there does not exist any phase shift between the x and y constituents of the electric field vector. If there does exist a phase shift δ between these constituents (δ≠kπ, k∈Z), the electric field vector experiences a more complex motion in the xy plane, which gives rise to circular polarization ($E_{0x}=E_{0y},\;\delta =\frac{\pi }{2}+k\pi ,\;k\in Z$) or elliptical polarization (otherwise). Vice versa, any polarization state can be built up from two mutually perpendicular linearly polarized components.

Further, it is instructive to delve somewhat into a brief theoretical description of the interaction between (polarized) light and matter. Absorption is an optical process whereby energy of an electromagnetic wave (a photon) is absorbed in a medium, accompanied by a transition of the atom/molecule in a higher energetic state. Depending on the photon energy and quantum states participating in the process, one can distinguish the infrared (IR) absorption, which is connected with a molecular transfer to a higher vibrational level within a given electronic state, and the UV/Vis absorption, which involves an electron excitation to a higher electronic state. In the context of this review, absorption will be mainly understood as the UV/Vis absorption. In order to restore the thermodynamic equilibrium, a molecule in an elevated electronic state can lose its energy and return to the ground state, which can be basically performed via one of the two processes: (i) The energy excess may dissipate in the form of heat (non-radiative crossing); or (ii) by a spontaneous emission of radiation, which is termed luminescence (fluorescence). Raman scattering belongs to the group of light scattering processes including annihilation of a primary photon and creation of a secondary photon with the frequency shifted from the primary photon by frequency of a molecular vibration. Thus, Raman scattering is a two-photon, inelastic process, which does not require the participating photons match the resonance frequency. For this reason, Raman scattering is a very weak process, but can be greatly amplified for molecules adsorbed in the vicinity of nanostructured metallic surfaces. This technique is called surface-enhanced Raman scattering (SERS) and it provides enhancement of the Raman scattering by a factor of 10^4^ or higher due to resonance excitation of plasmons localized in the metallic nanostructures [13]. Due to its high sensitivity, SERS has found use in numerous bio-related applications [14,15], e.g., for the detection of pharmaceuticals and drugs [16,17,18], food additives [19,20], explosives [21,22], disease biomarkers [23,24,25] and many others. 

Although “classical” models of the interaction between light and matter appeared already in the 1900s [12] (followed by a semi-classical theory of Raman scattering in 1930s by Placzek [26]), interaction of light with atoms or molecules cannot be fully described unless quantum theory comes into play. In the lowest degree of approximation, transition probability (to which intensity of a given spectral line is proportional) between two quantum states of an atom/molecule is governed by the Fermi’s golden rule. This rule says that in order for the transition to be allowed, the matrix element

(2) $$\langle 2|\hat{W}|1\rangle$$

must be different from zero. In Equation (2), |1〉 represents the wavefunction of the initial state, 〈2| the wavefunction of the final state and $\hat{W}$ is a perturbation operator, which usually corresponds to the presence of the electromagnetic wave.

In the case of absorption spectroscopy, the perturbation operator can be rewritten using the electric dipole moment of the molecule ($\vec{d}$) and the probability of the transition then derives from the transition dipole moment matrix element:

(3) $$\langle 2|\hat{d}|1\rangle .$$

In the case of Raman spectroscopy, the situation is more complex because in order to obtain the non-zero contribution, second-order perturbation theory has to be employed. The transition probability then derives from the second-rank tensor [27,28]:

(4) $$\alpha_{pq}^{12}=\underset{s\neq 1,2}{\sum }\left[\frac{\langle 2|{\hat{d}}_{p}|s\rangle \langle s|{\hat{d}}_{q}|1\rangle }{E_{1}-E_{s}+\hbar \omega_{i}}+\frac{\langle 2|{\hat{d}}_{q}|s\rangle \langle s|{\hat{d}}_{p}|1\rangle }{E_{2}-E_{s}-\hbar \omega_{i}}\right],$$

where $E_{1}$ and $E_{2}$ denote the energies of the initial (|1〉) and final (|2〉) quantum states of the molecule, $\hat{\vec{d}}$ is the electric dipole moment operator and the *s*-indices refer to all remaining quantum states of the molecule. ℏ denotes the reduced Planck’s constant and $\omega_{i}$ is the angular frequency of the incident radiation.

Equations (3) and (4) demonstrate basic differences between absorption and Raman measurements. While the molecular dipole moment is a vector quantity, the transition probability is expected to scale with the projection of the light polarization in the direction of the transition dipole moment of the molecule. However, Raman scattering is a two-photon process, which means that both the polarization of the incident beam as well as that of the scattered beam dictate the total Raman intensity. 

In the case of absorption spectroscopy, polarization effects in an ensemble of many randomly oriented molecules, such as liquid samples, are averaged out. That is because the total absorption intensity is given by spatial averaging over positions of all absorbing molecules. This can be computed explicitly in azimuthal coordinates:

(5) x=sinϑcosφ,  y=sinϑsinφ,  z=cosϑ

as:

(6) $$\langle {\left(\vec{E}\cdot \vec{d}\right)}^{2}\rangle ={\left(Ed\right)}^{2}\langle \cos^{2}\vartheta \rangle ={\left(Ed\right)}^{2}\frac{\int \cos^{2}\vartheta d\Omega }{\int d\Omega }$$

where dΩ=sinϑdϑdφ, ϑ∈(0,π) and φ∈(0, 2π) are standard azimuthal coordinates. After standard integration, we obtain:

(7) $$\langle \cos^{2}\vartheta \rangle =\frac{1}{3},$$

which implies that for many randomly oriented molecules, the absorbance does not depend on the direction of polarization. Since any polarization state can be built up from two mutually perpendicular linearly polarized components, absorbance of randomly oriented molecules never depends on the state of polarization including unpolarized (natural) radiation. This is not the case for molecules exhibiting some preferential orientation (linear dichroism) or phenomena beyond the dipole approximation in the case of chiral molecules (circular dichroism). Both of these effects are, however, beyond the scope of this paper. 

In the case of the Raman scattering, intensity of a given spectral line is given by a combination of the molecular Raman tensor and unit vectors of both the incident field ${\vec{e}}^{i}$ and the scattered field ${\vec{e}}^{sc}$:

(8) $$I\;\sim \;{\left(e_{p}^{sc}\alpha_{pq}^{12}e_{q}^{i}\right)}^{2}.$$

Directions ${\vec{e}}^{i}$ and ${\vec{e}}^{sc}$ are determined by the illumination-observation geometry, including the scattering angle and polarization of the incident beam (and possibly also polarization of the collected beam). Unlike in absorption spectroscopy, Raman measurements allow much wider choice of the experimental geometry including the polarization arrangement used as well as the scattering angle. Equation (8) naturally explains why the molecular quantity describing the disposition to Raman transition must intrinsically be of tensorial nature and also predicts that detected Raman intensities depend on the polarization arrangement used even in the case of liquids, i.e., randomly oriented molecules.

In both absorption and Raman measurements, molecular symmetry imposes certain restrictions on the number of vibrational modes being active or non-active in respective spectroscopic techniques. According to the number of symmetry elements characterizing a given isolated molecule, molecules can be classified into one out of 32 molecular point groups [29]. Having assigned a molecule to a point group, the methods of group theory allow one to decompose the vibrational signature of the molecule into so-called irreducible representations. The number of normal modes belonging to each irreducible representation (out of the 3N−6 normal modes in total) is calculated using a well-established algorithm, making use of character tables of molecular point groups [29,30]. For the purpose of spectroscopy, it is important to note that each irreducible representation may transform as a particular linear or quadratic function of coordinates. Since the constituents of the electric dipole moment have the same transformation properties as x, y and z and elements of a Raman tensor as $x^{2},\;y^{2},\;z^{2},\;xy,\;xz$ and yz, it is relatively easy to predict which vibrations will be active in Raman/IR absorption spectra: In order for a given vibrational mode to be allowed in IR absorption spectra, its irreducible representation must span at least one of x, y and z species (in the case of randomly-oriented molecules). For the transition to be allowed in Raman spectra, its irreducible representation must span at least one of the quadratic ($x^{2},\;y^{2},\;z^{2},\;xy,\;xz$ and yz) species (again in the case of randomly-oriented molecules).

## 3. Polarization and Angular Dependences in Raman Spectroscopy

### 3.1. Derivation of Raman Intensities as a Function of the Polarization and Angular Arrangement

**Figure 1:**
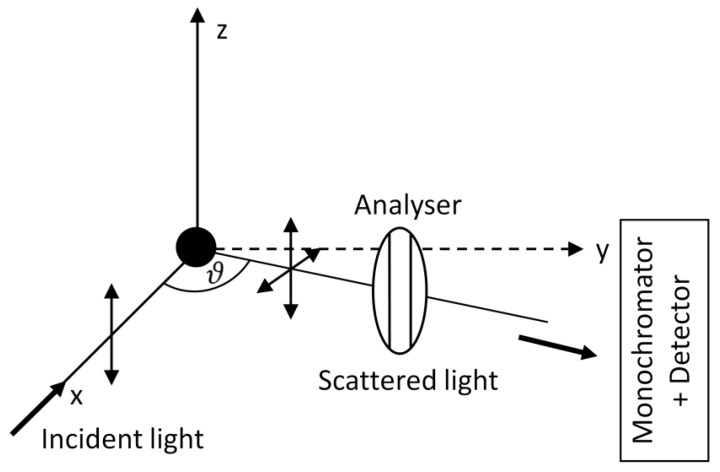
Scheme of a general Raman scattering geometry. The excitation beam travels along the x direction with polarization in the z direction and the detector collects light scattered in the xy plane at an angle ϑ with respect to the x axis. An analyser may be inserted between the sample and the monochromator to allow only one specific Raman polarization enter the detector.

Let us consider a general experimental geometry for Raman scattering measurements, depicted in Figure 1. Let us suppose that a Cartesian coordinate system is chosen so that the sample is placed in its origin, the excitation beam travels along the x direction with polarization along the z direction and the detector is oriented to collect light scattered in the xy plane at an angle ϑ with respect to the x axis. The angle ϑ is termed the scattering angle and it can take any value from 0° to 180°. The xy plane is termed the scattering plane. In the following, we will refer to the z components of the electric field as to the normal or vertical (with respect to the scattering plane, denoted as v or ⏊) and the components lying in the scattering plane as to the horizontal (denoted as h or ||). In general, the scattered light contains both vertical and horizontal components—that is why an analyser is often inserted between the monochromator and the sample to distinguish these two polarizations. In order to overcome different grating responses for different light polarizations, a scrambler must be inserted between the analyser and the monochromator (not shown in Figure 1 for simplicity). 

With the fixed angle ϑ, 4 different polarization combinations can be thought of to retrieve Raman spectra: There are two basic possibilities of setting the polarization of the incident beam (either perpendicular to the scattering plane as depicted in Figure 1, i.e., in the z direction, or lying in the scattering plane, i.e., in the y direction) as well as two basic possibilities of rotating the analyser to pick out either horizontal or vertical polarization. Thus, we adopt the symbols:

(9) $$I\left(\vartheta ,{⏊}^{sc},{⏊}^{i}\right)=I_{vv},\;I\left(\vartheta ,{\|}^{sc},{⏊}^{i}\right)=I_{vh},\;I\left(\vartheta ,{⏊}^{sc},{\|}^{i}\right)=I_{hv},\;I\left(\vartheta ,{\|}^{sc},{\|}^{i}\right)=I_{hh}$$

to account for each of these polarization arrangements. The first symbol in each bracket denotes the scattering angle, the second symbol refers to polarization of the scattered beam (with respect to the scattering plane) and the third one refers to polarization of the incident beam. Sometimes the scattering angle is unimportant and as a shorthand we introduce the notation $I_{vv}$, $I_{vh}$, $I_{hv}$ and $I_{hh}$ with the first subscript standing for polarization of the incident beam and the latter standing for polarization of the scattered beam. Our task is to derive expressions for intensities of a given Raman line, measured under respective arrangements. It is important to note that for ϑ=0° or ϑ=180° the scattering plane is not well-defined. Nevertheless, we will show that the general considerations above will become trivial in these special cases. 

Equation (8) says that each of the 4 possible polarization arrangements mentioned above picks out different elements of the Raman tensor of the molecules. In other words, specific illumination-observation geometry determines which elements of the Raman tensor will appear in the final expressions dictating the intensity of a given Raman line. The Cartesian components of the electric field vector of the scattered beam are:

(10) $$E_{x}^{sc}=E\left(\vartheta ,{\|}^{sc}\right)\sin\vartheta ,\;E_{y}^{sc}=E\left(\vartheta ,{\|}^{sc}\right)\cos\vartheta ,\;E_{z}^{sc}=E\left(\vartheta ,{⏊}^{sc}\right),$$

where $E\left(\vartheta ,{\|}^{sc}\right)$ and $E\left(\vartheta ,{⏊}^{sc}\right)$ denote components of the amplitude of the scattered beam lying in the scattering plane and in the direction perpendicular to the scattering plane, respectively. By combination of Equations (8)–(10), the following explicit formulas can be obtained (for simplicity, we further omit the upper indices 12 in denomination of the molecular Raman tensor α):

(11) $$I\left(\vartheta ,{⏊}^{sc},{⏊}^{i}\right)\;\sim \;\alpha_{zz}^{2},\;I\left(\vartheta ,{\|}^{sc},{⏊}^{i}\right)\;\sim \;{\left(\alpha_{xz}\sin\vartheta +\alpha_{yz}\cos\vartheta \right)}^{2},$$

(12) $$I\left(\vartheta ,{⏊}^{sc},{\|}^{i}\right)\;\sim \;\alpha_{zy}^{2},\;I\left(\vartheta ,{\|}^{sc},{\|}^{i}\right)\;\sim \;{\left(\alpha_{xy}\sin\vartheta +\alpha_{yy}\cos\vartheta \right)}^{2}.$$

In the case of an ensemble of randomly oriented molecules, respective squares or Raman tensor elements in Equations (11) and (12) are to be replaced with their spatially-averaged values as contributions from each individual molecule sum up incoherently. This can be performed in a manner analogous to Equation (6), however, molecular polarizability responsible for the Raman scattering is a tensor rather than a vector quantity, which makes the determination of spatially-averaged tensor elements a more tedious procedure. Any spatial rotation of the molecule with respect to a given reference frame can be expressed unambiguously by a rotational matrix associated with 3 Euler angles, whose elements must be averaged in a manner analogous to Equation (6) [31]. An alternative and less time-consuming approach involves computation of isotropic averages of respective products of direction cosines between two Cartesian axis systems (again only 3 out of 9 direction cosines are independent) [27,32]. For an ensemble of many randomly oriented molecules, intensity of a given Raman line in a given experimental geometry may be expressed as a linear combination of Raman tensor invariants. These are usually referred to as:

The square of the mean polarizability a:

(13) $$a^{2}=\frac{{\left(\alpha_{xx}+\alpha_{yy}+\alpha_{zz}\right)}^{2}}{9},$$

the anisotropy γ:

(14) $$\gamma^{2}=\frac{{\left(\alpha_{xx}-\alpha_{yy}\right)}^{2}+{\left(\alpha_{yy}-\alpha_{zz}\right)}^{2}+{\left(\alpha_{zz}-\alpha_{xx}\right)}^{2}}{2}+3\frac{{\left(\alpha_{xy}+\alpha_{yx}\right)}^{2}+{\left(\alpha_{yz}+\alpha_{zy}\right)}^{2}+{\left(\alpha_{zx}+\alpha_{xz}\right)}^{2}}{4},$$

and the antisymmetric anisotropy δ:

(15) $$\delta^{2}=3\frac{{\left(\alpha_{xy}-\alpha_{yx}\right)}^{2}+{\left(\alpha_{yz}-\alpha_{zy}\right)}^{2}+{\left(\alpha_{zx}-\alpha_{xz}\right)}^{2}}{4}.$$

For non-resonance Raman scattering only the isotropic invariant a and the anisotropic invariant γ are relevant since in this case the Raman tensor is symmetric and the antisymmetric invariant δ is identically zero. The result of the averaging procedure is [27,32]:

(16) $$\langle \alpha_{xx}^{2}\rangle =\langle \alpha_{yy}^{2}\rangle =\langle \alpha_{zz}^{2}\rangle =\frac{45a^{2}+4\gamma^{2}}{45},\;\;\langle \alpha_{xy}^{2}\rangle =\langle \alpha_{xz}^{2}\rangle =\langle \alpha_{yz}^{2}\rangle =\frac{3\gamma^{2}+5\delta^{2}}{45},$$

(17) $$\langle \alpha_{xz}\alpha_{yz}\rangle =\langle \alpha_{xy}\alpha_{yy}\rangle =0.$$

This result shows the intuitive notion that the average values of all diagonal elements of the Raman tensor are equal and also all off-diagonal elements are equal, irrespective of a specific choice of the system of coordinates. Also all Raman tensor products which involve one common subscript are zero. Putting these values in Equations (11) and (12) yields:

(18) $$I\left(\vartheta ,{⏊}^{sc},{⏊}^{i}\right)\;\sim \;\frac{45a^{2}+4\gamma^{2}}{45},\;I\left(\vartheta ,{\|}^{sc},{⏊}^{i}\right)\;\sim \;\frac{3\gamma^{2}+5\delta^{2}}{45},$$

(19) $$I\left(\vartheta ,{⏊}^{sc},{\|}^{i}\right)\;\sim \;\frac{3\gamma^{2}+5\delta^{2}}{45},\;\;\left(\vartheta ,{\|}^{sc},{\|}^{i}\right)\;\sim \;\frac{45a^{2}+4\gamma^{2}}{45}\cos^{2}\vartheta +\frac{3\gamma^{2}+5\delta^{2}}{45}\sin^{2}\vartheta .$$

Equations (18) and (19) reveal that actually the only polarization arrangement in which the intensity of a given line will depend on the scattering angle ϑ is the arrangement in which both the incident beam as well as the scattered beam are polarized in the scattering plane. 

In order to avoid misunderstanding, it is important to realize that any combination of Raman tensor invariants (13)–(15) is again an invariant. In literature, many alternative definitions of Raman tensor invariants may be found [27]. For example, Raman tensor invariants used by Placzek [26] are:

(20) $$G^{0}=3a^{2},\;\;G^{a}=\frac{2}{3}\delta^{2},\;\;G^{s}=\frac{2}{3}\gamma^{2}.$$

A quantity which can be very easily measured experimentally is the Raman depolarization ratio, which is defined as the ratio of Raman intensities measured under two selected configurations. Most commonly, the depolarization ratio ρ is assumed as the ratio between the intensity of the Raman field polarized orthogonal to the incident field and the intensity of the Raman field polarized parallel to the laser field [27,28]. The most common way of retrieving the depolarization ratio is fixing the incident polarization and rotating the analyser. In our notation, this yields to:

(21) $$\rho \left(\vartheta ,{⏊}^{i}\right)=\frac{I\left(\vartheta ,{\|}^{sc},{⏊}^{i}\right)\;}{I\left(\vartheta ,{⏊}^{sc},{⏊}^{i}\right)}=\frac{I_{vh}}{I_{vv}}.$$

Although many textbooks resort only to the definition of the depolarization ratio given by Equation (21) [28,30], this formula can be easily generalized and many alternative definitions of the depolarization ratio can be found in the literature [27], such as:

(22) $$\rho \left(\vartheta ,{\|}^{i}\right)=\frac{I\left(\vartheta ,{⏊}^{sc},{\|}^{i}\right)}{I\left(\vartheta ,{\|}^{sc},{\|}^{i}\right)}=\frac{I_{hv}}{I_{hh}},\;\;\rho \left(\vartheta ,{⏊}^{sc}\right)=\frac{I\left(\vartheta ,{⏊}^{sc},{\|}^{i}\right)\;}{I\left(\vartheta ,{⏊}^{sc},{⏊}^{i}\right)}=\frac{I_{hv}}{I_{vv}},$$

(23) $$\rho \left(\vartheta ,{⏊}^{sc},{⏊}^{i},{\|}^{sc},{\|}^{i}\right)=\frac{I\left(\vartheta ,{⏊}^{sc},{⏊}^{i}\right)\;}{I\left(\vartheta ,{\|}^{sc},{\|}^{i}\right)}=\frac{I_{vv}}{I_{hh}},$$

etc. The depolarization ratio provides unique information on the Raman polarizability tensor of a given molecular vibration and is therefore a very useful tool. Indeed, in the case of randomly oriented molecules, we have:

(24) $$\rho \left(\vartheta ,{⏊}^{i}\right)=\frac{I_{vh}\;}{I_{vv}}=\frac{3\gamma^{2}+5\delta^{2}}{45a^{2}+4\gamma^{2}}.$$

Similar formulas can be derived for the depolarization ratios given by Equations (22) and (23). It is obvious that the introduction of the depolarization ratios is very convenient because it allows to exclude the insignificant multiplicative constant relating Raman intensities to specific combinations of squares of Raman tensor elements. The depolarization ratio defined by Equation (24) is usually termed the molecular depolarization ratio since it is an intrinsic property of the Raman probe (contrary to the depolarization ratio measured in presence of smooth/nanostructured metallic surfaces where the mutual interaction between light/analyte/metal substantially affect the measured depolarization ratio). From Equation (24) it immediately follows that for non-resonance Raman scattering (δ=0) the molecular depolarization ratio is always bound between 0 and 3/4. A specific value of ρ will depend on the symmetry of the vibration involved. For totally symmetric vibrations $\gamma^{2}=0$ and the depolarization ratio tends to zero. Conventionally, such a Raman line is said to be completely polarized. This aspect can be explained straightforwardly from a classical point of view: For totally symmetric vibrations, the polarizability tensor will become a scalar and the direction of the oscillating dipole will match the direction of the incident field polarization. Since total Raman intensity is proportional to the scalar product of the oscillating dipole moment and the detected field, one would expect that when setting the analyser parallel to the direction of the oscillating dipole (the incident polarization), the intensity will reach its maximum, while after rotating the analyser about 90° the intensity will drop to its minimum. On the other hand, for non-totally symmetric vibrations $a^{2}=0$ and the depolarization ratio is 3/4 [27,28]. Conventionally, such a Raman line is said to be depolarized. When 0<ρ< 3/4, the Raman line is said to be (partially) polarized [27].

**Figure 2:**
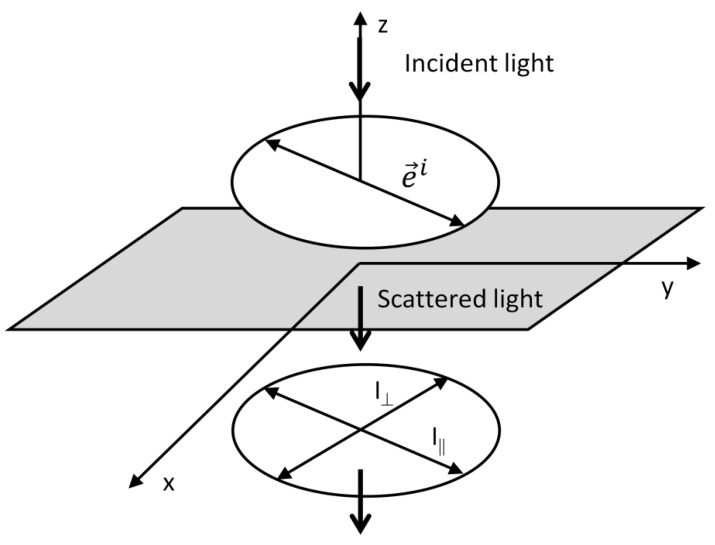
Scheme of a forward-scattering geometry. Linearly polarized light is falling on the plane of the sample (xy) from above. Analyser can be set to retrieve either a perpendicular component of the laser field ($I_{⏊}$) or a parallel one ($I_{\|}$). Analogous situation occurs in the case of a backscattering geometry.

Previous considerations are valid for any angle ϑ apart from ϑ=0° (forward scattering) and ϑ=180° (backscattering). In this instance the scattering plane is not well-defined and the symbols ${⏊}^{sc},{⏊}^{i},{\|}^{sc}$ and ${\|}^{i}$ as well as $I_{vv}$, $I_{vh}$, $I_{hv}$ and $I_{hh}$ lose their sense. Nevertheless, the situation is trivial in this case. Let us resketch the experimental geometry in Figure 2, adopting the direction of the z axis parallel to the wavevector of the incident (scattered) light. After illuminating the sample with light with a given polarization, confined to the xy plane, the analyser may be set to collect Raman field polarized parallel or orthogonal to the laser field. Alternatively, Raman measurements may be performed with a fixed position of the analyser and varying polarization of the incident beam. Respective arrangements lead to probing of the averages of the squares of the diagonal/off-diagonal Raman tensor elements. In this case, the z components of the Raman tensor do not figure in the equations. Formally, the 4 respective polarization combinations discussed above split into two groups since $I\left(0{}^{\circ},{⏊}^{sc},{⏊}^{i}\right)=I\left(0{}^{\circ},{\|}^{sc},{\|}^{i}\right)$ and $I\left(0{}^{\circ},{\|}^{sc},{⏊}^{i}\right)=I\left(0{}^{\circ},{⏊}^{sc},{\|}^{i}\right)$ in this case, the same holding also for ϑ=180°. The depolarization ratio for ϑ=0° or ϑ=180° can be expressed simply as the ratio of intensities obtained with crossed polarizations to intensities obtained with parallel polarizations. Formally:

(25) $$\rho =\frac{I\left(0{}^{\circ}\;\mathrm{or}\;180{}^{\circ},{\|}^{sc},{\|}^{i}\right)}{I\left(0{}^{\circ}\;\mathrm{or}\;180{}^{\circ},{\|}^{sc},{\|}^{i}\right)},$$

which takes the form given by Equation (24) in the case of randomly oriented molecules. 

Further, let us inspect another widely-used geometry in which ϑ=90° (right-angle geometry). After inserting ϑ=90° in Equation (19), we obtain:

(26) $$I\left(90{}^{\circ},{\|}^{sc},{⏊}^{i}\right)=I\left(90{}^{\circ},{⏊}^{sc},{\|}^{i}\right)=I\left(90{}^{\circ},{\|}^{sc},{\|}^{i}\right)\neq I\left(90{}^{\circ},{⏊}^{sc},{⏊}^{i}\right),$$

(27) $$I_{vh}=I_{hv}=I_{hh}\neq I_{vv}.$$

It means that for retrieving information on the Raman depolarization ratio (and consequently on the symmetry of the vibration involved), $I_{vv}$ and any of $I_{vh}$, $I_{hv}$ and $I_{hh}$ are required.

### 3.2. Simple Illustration of the Depolarization Ratio Measurement

**Figure 3:**
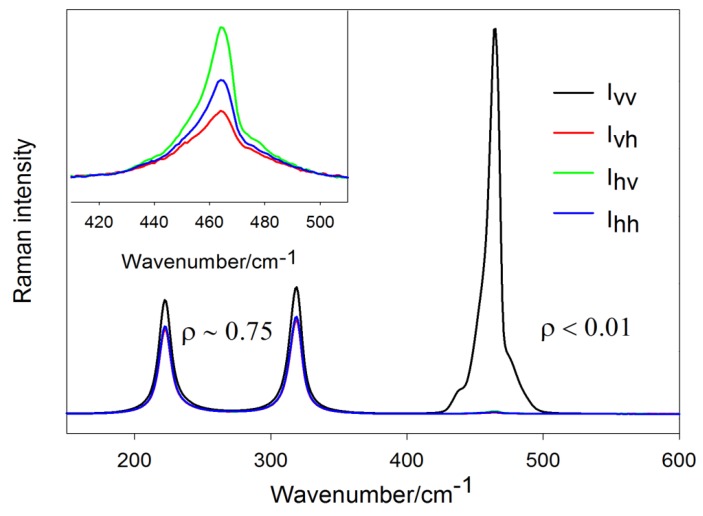
Polarized Raman spectra of CCl_4_. The excitation wavelength was 532 nm, laser power 100 mW and the accumulation time 1 min. No further corrections have been made.

In this paragraph, we demonstrate previous considerations on a simple example of liquid CCl_4_ measured in our laboratory in the right-angle geometry (excitation wavelength 532 nm). The default polarization of the incident beam in this experimental setup is perpendicular to the scattering plane, but may be rotated by 90° using a half-wave plate or a Fresnel rhomb retarder. The CCl_4_ is a highly symmetric molecule belonging to the $T_{d}$ point group. Polarized Raman spectra obtained in all four polarization arrangements are displayed in Figure 3.

Analysis of respective depolarization ratios suggests that the band centered around 465 cm^−1^ can be classified as totally symmetric (the “disrupted” structure of the 465-cm^−1^ band is owed to different Cl isotopes which lift the degeneracy of this peak) while the bands around 222 and 319 cm^−1^ as non-totally symmetric modes. Indeed, group theory predicts the $\Gamma^{3N-6}$ representation of CCl_4_ to be $A_{1}⊕E⊕2F_{2}$ (E being doubly degenerate and F being triply degenerate, making 3·5−6=9 vibrational modes in total). 

Basically, those values obtained from the experiment confirm that $I_{vh}$, $I_{hv}$ and $I_{hh}$ configurations are equivalent. However, closer analysis (inset of Figure 3) reveals slight differences in respective polarization arrangements, which is obviously attributable to transfer optics imperfections. The depolarization ratios obtained from measurements with varying incident polarization are larger than from the measurements where the incident polarization was kept constant, which shows some subtle imperfection of the half-wave plate, transmitting a small fraction of orthogonal polarization as well (making the relative error particularly large for highly polarized lines where ρ≈0). Thus, polarization-dependent Raman spectra of CCl_4_ serve well for checking the correct function of the scrambler and other optical components used for more complex polarization-dependent measurements.

Last but not least, the literature is not consistent about whether peak heights (above spectral background) or rather integrated areas should actually enter Equations (21)–(23). In many instances (such as in the case of CCl_4_) these two ways produce almost identical results as the line width is not expected to change with polarization. On the other hand, in the case of several overlapping bands, determination of the spectral background and delimiting the peak area may be difficult and the two methods may give somewhat different results. In these cases, fitting the spectrum with the sum of the Lorentz curves would be advisable.

### 3.3. Other Possible Polarization Arrangements Used

Previous survey of the most widely-used geometries for the depolarization ratio measurements is still not exhaustive. All other possible geometries can be resolved exploiting the fact that any polarization state can be decomposed into a superposition of two independent, linearly polarized waves with their electric field vectors perpendicular to each other. Other possible experimental geometries (maybe even the most frequent) involve the situations when no analyser is used, i.e., the excitation field is either vertical or horizontal, but polarization of the detected field is not characterized (or a far less common case when the excitation field is unpolarized and respective scattered field components are detected). For the sake of completeness, we adopt the symbols:

(28) $$I\left(\vartheta ,n^{sc},{⏊}^{i}\right)=I\left(\vartheta ,{⏊}^{sc},{⏊}^{i}\right)+I\left(\vartheta ,{\|}^{sc},{⏊}^{i}\right),$$

(29) $$I\left(\vartheta ,n^{sc},{\|}^{i}\right)=I\left(\vartheta ,{⏊}^{sc},{\|}^{i}\right)+I\left(\vartheta ,{\|}^{sc},{\|}^{i}\right),$$

and the less commonly used symbols:

(30) $$I\left(\vartheta ,{⏊}^{sc},n^{i}\right)=I\left(\vartheta ,{⏊}^{sc},{⏊}^{i}\right)+I\left(\vartheta ,{⏊}^{sc},{\|}^{i}\right),$$

(31) $$I\left(\vartheta ,{\|}^{sc},n^{i}\right)=I\left(\vartheta ,{\|}^{sc},{⏊}^{i}\right)+I\left(\vartheta ,{\|}^{sc},{\|}^{i}\right).$$

Equations (28) and (29) show that investigating a total Raman intensity without an analyser is equivalent to performing two experiments in a row: setting the analyser to collect the horizontal polarization, then rotating the analyser by 90° to collect the vertical polarization and summing up both results. An analogous situation is true for Equations (30) and (31). Moreover, respective left-hand sides of Equations (28)–(31) can be combined and yield further alternative depolarization ratio definitions. Surprisingly, information on the orientation-averaged squares of the Raman tensor elements (and consequently information on the symmetry of molecular vibrations involved) can be extracted even without an analyser—all that one needs to do is to record the Raman spectra with the incident field polarization perpendicular to the scattering plane and parallel to the scattering plane, respectively. Let us, for instance, determine the expected depolarization ratio in the right-angle geometry with varying incident polarization and no analyser used. By combining Equations (18), (19), (28) and (29), we obtain:

(32) $$\rho \left(90{}^{\circ},n^{sc}\right)=\frac{I\left(90{}^{\circ},n^{sc},{\|}^{i}\right)}{I\left(90{}^{\circ},n^{sc},{⏊}^{i}\right)}=\frac{6\gamma^{2}+10\delta^{2}}{45a^{2}+7\gamma^{2}+5\delta^{2}}.$$

The depolarization ratio for non-resonance Raman scattering ($\delta^{2}=0)$, defined in the manner of Equation (32), is always bound between 0 and 6/7.

### 3.4. Directional Properties in Raman Spectroscopy

**Figure 4:**
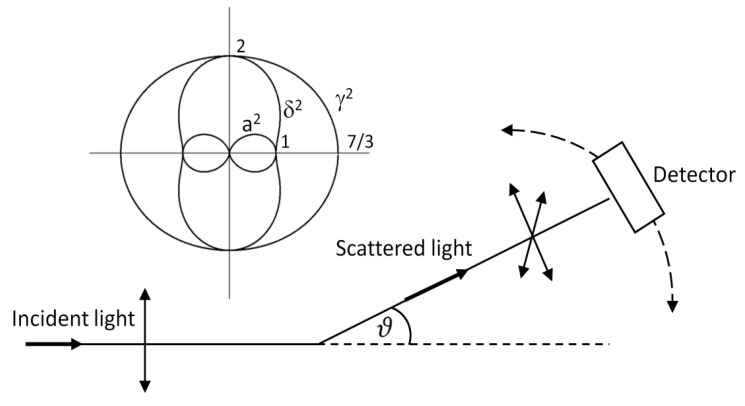
Angular dependence of the Raman signal. When both the incident and scattered fields are polarized parallel to the scattering plane or when one field is polarized parallel and the latter is unpolarized, the detected Raman intensity will be angular-dependent. Angular dependences for molecules with a dominant contribution of $a^{2}$, $\gamma^{2}$ and $\delta^{2}$ respectively when moving the detector by an angle ϑ from 0° to 360° and no analyser is used are indicated in the inset.

At the end of this section, implications of previous considerations on the angular dependences in Raman spectroscopy will be discussed. We have already seen that out of the 4 basic polarization arrangements, the only one where the Raman intensity actually depends on the angle ϑ is the $I\left(\vartheta ,{\|}^{sc},{\|}^{i}\right)$ arrangement (Equation (19)). Thus, let us resketch the Raman measurement scheme as in Figure 4. Supposing the scattering process takes place in the plane of the sheet, different Raman intensities are expected to arise when moving the detector about the axis perpendicular to the scattering plane. This intensity modulation occurs only if both the excitation and the detected field are polarized parallel to the scattering plane, or when the detected field is unpolarized (or a far less common case when the excitation field is unpolarized and parallel field component is detected). If at least one of the excitation/detected field components is perpendicular to the scattering plane, Raman intensity will no longer be the function of ϑ as follows directly from Equation (18). By contrast, if both the excitation and detected fields were perpendicular to the plane of the sheet, Raman intensity would be modulated when moving the detector in the plane perpendicular to the sheet.

Let us explore in more detail the case when the excitation line is polarized parallel to the scattering plane and polarization of the scattered beam is not characterized, i.e., the analyser is missing. Again, we will suppose that the Raman scatterers are randomly oriented molecules. In our notation:

(33) $$I\left(\vartheta ,n^{sc},{\|}^{i}\right)=I\left(\vartheta ,{⏊}^{sc},{\|}^{i}\right)+I\left(\vartheta ,{\|}^{sc},{\|}^{i}\right)\;\sim \;\frac{3\gamma^{2}+5\delta^{2}}{45}+\frac{45a^{2}+4\gamma^{2}}{45}\cos^{2}\vartheta +\frac{3\gamma^{2}+5\delta^{2}}{45}\sin^{2}\vartheta .$$

Rearranging Equation (33), we obtain:

(34) $$I\left(\vartheta ,n^{sc},{\|}^{i}\right)\;\sim \;a^{2}\cos^{2}\vartheta +\frac{\gamma^{2}}{45}\left(6+\cos^{2}\vartheta \right)+\frac{\delta^{2}}{9}\left(1+\sin^{2}\vartheta \right).$$

Angular dependence of the three terms on the right-hand side of Equation (34) is displayed in the inset of Figure 4. From here it follows that the angular dependence of a totally symmetric vibration (a≠0, γ=0, δ=0) is dictated by the $\cos^{2}\vartheta$ function, i.e., the same as encountered in investigating the angular dependence of the dipole radiation in classical physics [12]. For a vibration whose dominant invariant is $\gamma^{2}$, the detected intensity will only slightly depend on the angle ϑ via the term $6+\cos^{2}\vartheta$. Finally, in the case of resonance Raman scattering, the anisotropic contribution will scale as $1+\sin^{2}\vartheta$. It is the only term which experiences its maximum for ϑ=90° (anomalous angular dependence). 

## 4. Polarization Effects for Crystals and Molecules Adsorbed on Smooth Surfaces

We have demonstrated that Raman scattering measurements of systems consisting of freely rotating molecules allow retrieving only spatially-averaged values of respective squares of Raman tensor elements. However, more detailed information can be extracted from anisotropic systems such as single crystals [33] or molecules adsorbed on planar surfaces possessing some sort of preferential orientation. In single crystals, all molecular axes are lined up within the unit cell in the same direction for each cell. Under these circumstances, polarization of the incident light set to ${\vec{e}}_{q}$ and polarization of the scattered light set to ${\vec{e}}_{p}$ will pick out only the $\alpha_{pq}$ component of the Raman tensor (see Equation (8)). Thus, it is possible to analyse each of the Raman tensor elements separately by employing different polarization arrangements. According to Porto’s notation, each configuration for Raman scattering measurements of single crystals is unambiguously described by four symbols, such as i(jk)l (where i,j,k,l=x,y,z). The symbols inside the parentheses are, left to right, the polarization of the incident and the scattered light, the leftmost symbol denotes the wavevector of the incident light and the rightmost symbol the wavevector of the scattered light [33]. However, in the case of anisotropic systems such as single crystals, the selection rules are a bit stricter in some sense. In the geometry determined by the Porto’s notation i(j,k)l, only those vibrations covering the same irreducible representation as jk (j,k=x,y,z) will be allowed. As a consequence, symmetry species of single crystals of known orientation may be distinguished with the use of polarized radiation. In this paper, we will not deal further with crystal samples, but we will demonstrate how exploiting the formalism of the group theory together with experimental observation can determine the orientation of molecules on (whether planar or rough) surfaces.

In 1982, Moskovits coined the term “surface selection rules” to account for modification of the absorption, emission and Raman scattering intensities of molecules in the vicinity of planar metallic surfaces [34,35], although some considerations concerning variation in near-field intensities with the angle of incidence/polarization had been made before [4,5]. A year later, Creighton derived expressions enabling him to determine the orientation of molecules adsorbed at the surface of a small metallic sphere [36]. Both results show that depending on the molecular orientation with respect to the surface, differences in the relative enhancement of modes belonging to different irreducible representations (symmetry classes) may be expected. Let us first consider the situation on a flat surface and proceed further to molecules adsorbed on plasmonic nanostructures. The latter is a more complicated case due to the fact that in this instance both the symmetry of the Raman tensor and coupling efficiency of the laser light to nanostructures influence the final spectral pattern.

What lies behind the surface selection rules for molecules adsorbed on planar (metallic) surfaces is the fact that the field felt by the analyte may be viewed as a superposition of the incident wave and the reflected wave. In principle, this interference can be both constructive and destructive and the total intensity of a given optical process will depend, among other things, on reflectivity of the substrate (and thus wavelength, of which reflectivity is a function), angle of incidence and polarization. For molecules adsorbed on planar surfaces, the surface selection rules may be interpreted simply in the framework of the Fresnel coefficients. Briefly, when light in vacuum (air) is incident on a planar interface between two different media, the latter possessing a (complex) refraction index $\tilde{n}=n+ik$, it is common procedure to resolve the electric field vectors of both the incident and the reflected wave into two orthogonal components. Assuming that the interface lies in the xy plane, the plane of incidence is yz and light of frequency ω falls on the interface at an angle ϑ with respect to the surface normal, the wavevectors of both the incident and the reflected light may be expressed as:

(35) $${\vec{k}}_{i}=\frac{\omega }{c}\left(0,\;\sin\vartheta ,\;-\cos\vartheta \right),\;\;{\vec{k}}_{r}=\frac{\omega }{c}\left(0,\;\sin\vartheta ,\;\cos\vartheta \right),$$

and their electric field vectors as:

(36) $${\vec{E}}_{i}=(E_{i}^{s},\;E_{i}^{p}\cos\vartheta ,\;E_{i}^{p}\sin\vartheta ),$$

(37) $${\vec{E}}_{r}=\left(E_{r}^{s},\;-E_{r}^{p}\cos\vartheta ,\;E_{r}^{p}\sin\vartheta \right)=(r_{s}E_{i}^{s},\;-r_{p}E_{i}^{p}\cos\vartheta ,\;r_{p}E_{i}^{p}\sin\vartheta ),$$

where the amplitude of the electric field vector of the incident beam projected on the plane of incidence is denoted as $E_{i}^{p}$ and on the direction perpendicular to the plane of incidence as $E_{i}^{s}$, similarly $E_{r}^{p}$ and $E_{r}^{s}$ are amplitudes of the electric field vector of the reflected beam projected on the plane of incidence and on the direction perpendicular to the plane of incidence, respectively. $r_{s}$ and $r_{p}$ are the Fresnel reflection coefficients for s- and p- polarized light. By applying boundary conditions to the solutions of Maxwell’s equations, it can be shown that [12,13]:

(38) $$r_{s}=\frac{\cos\vartheta -\sqrt{{\tilde{n}}^{2}-\sin^{2}\vartheta }}{\cos\vartheta +\sqrt{{\tilde{n}}^{2}-\sin^{2}\vartheta }},\;r_{p}=\frac{{\tilde{n}}^{2}\cos\vartheta -\sqrt{{\tilde{n}}^{2}-\sin^{2}\vartheta }}{{\tilde{n}}^{2}\cos\vartheta +\sqrt{{\tilde{n}}^{2}-\sin^{2}\vartheta }}.$$

In general, both Fresnel reflection coefficients become complex, thus $r_{s}\equiv {\tilde{r}}_{s}$ and $r_{p}\equiv {\tilde{r}}_{p}$. Interference between the incident and the reflected beam at the interface suggests that the electric field intensity felt by the adsorbed molecules has the components:

(39) $$E_{x}=E_{i}^{s}\left(1+r_{s}\right),\;\;E_{y}=E_{i}^{p}\cos\vartheta \left(1-r_{p}\right),\;\;E_{z}=E_{i}^{p}\sin\vartheta \left(1+r_{p}\right).$$

**Figure 5:**
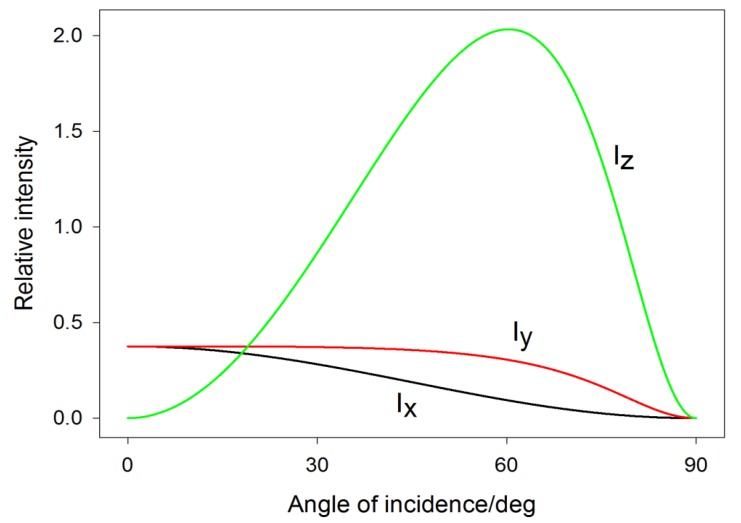
Calculated near-field intensities in the vicinity of a smooth Ag surface for an arbitrarily polarized light wave, λ=500 nm. Calculations were performed for different angles of incidence and the refractive index ${\tilde{n}}_{Ag}=0.05+3.09i$ [38].

Since the real part of $r_{s}$ is negative and the real part of $r_{p}$ is usually positive, it is clear that only the z (normal) component of the electric field at the surface will benefit from constructive interference, whereas the other two (tangential) components exhibit rather destructive interference. For most metals, $r_{s}$ approaches −1 and $r_{p}$ approaches 1 in the IR and the visible spectral region at angles around ~60°. For example, for silver, light intensity associated with the field in the z direction ($E_{z}^{2}$) can be as much as ~6× stronger than the intensity in other directions ($E_{x}^{2}+E_{y}^{2}$) around the wavelength of 500 nm (Figure 5). The difference tends to be even more pronounced in the IR [37]. 

Since IR absorption intensity derives from the scalar product of the transition dipole moment and direction of light polarization, one can deduce that the IR absorption by molecular vibrations with a nonzero component of the transition dipole moment perpendicular to the surface will be strengthened, whereas the absorption by vibrations with the only nonzero component parallel to the surface will be weakened with respect to a “free” molecule [37]. This observation together with the aid of the group theory becomes useful when aiming to elucidate molecular orientation on the surface. Let us illustrate this aspect with a simple example. For each individual molecule, a local Cartesian system of coordinates $x^{\prime },\;y^{\prime }\;z^{\prime }$ may be introduced to describe its orientation in space (with $z^{\prime }$ usually being the principal axis of symmetry). Providing that the molecule binds to the surface so that $x=x^{\prime },\;y=y^{\prime }$ and $z=z^{\prime }$, the strongest modes observed in the IR spectrum will be those belonging to the irreducible representation with the same transformation properties as z. By contrast, in case the molecule is positioned on the surface so that its $z^{\prime }$ axis lies parallel to the surface, the modes spanning z coordinate are expected to be very weak. 

**Figure 6:**
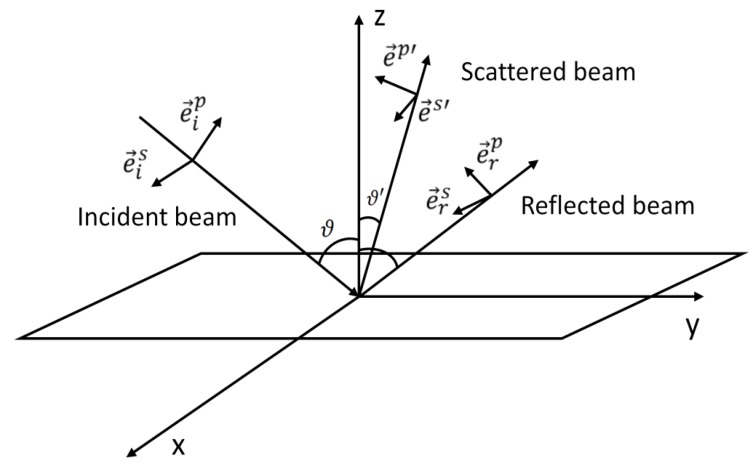
Scheme of the geometrical layout and definition of the coordinates. All symbols have usual meanings as described in the text. The components of the scattered field experiencing a reflection from the surface (${\vec{E}}_{r}^{s{}^{\prime }}$ and ${\vec{E}}_{r}^{p{}^{\prime }}$) are not included in the scheme for simplicity.

In the case of Raman scattering of molecules adsorbed on flat surfaces, a very similar principle applies in the case of the scattered radiation. Providing that the scattered radiation is collected at an arbitrary angle $\vartheta {}^{\prime }$ as shown in Figure 6, the wavevector of the scattered light will be:

(40) $${\vec{k}}_{sc}=\frac{\omega }{c}\left(0,\;\sin\vartheta {}^{\prime },\;\cos\vartheta {}^{\prime }\right),$$

the directly scattered laser field is:

(41) $${\vec{E}}_{sc}=(E_{sc}^{s},\;-E_{sc}^{p}\cos\vartheta^{\prime },\;E_{sc}^{p}\sin\vartheta {}^{\prime }),$$

and the contribution to the total detected field experiencing a single reflection from the surface will be:

(42) $$(r_{s}^{\prime }E_{sc}^{s},\;r_{p}^{\prime }E_{sc}^{p}\cos\vartheta^{\prime },\;r_{p}^{\prime }E_{sc}^{p}\sin\vartheta {}^{\prime }).$$

Thus, the total detected field will have the components:

(43) $$\;E_{x}=E_{sc}^{s}\left(1+r_{s}^{\prime }\right),\;\;E_{y}=E_{sc}^{p}\cos\vartheta {}^{\prime }\left(r_{p}^{\prime }-1\right),\;\;E_{z}=E_{sc}^{p}\sin\vartheta {}^{\prime }\left(1+r_{p}^{\prime }\right).$$

As usual, $E_{sc}^{p}$ and $E_{sc}^{s}$ denote the amplitudes of the electric field vector of the scattered radiation projected on the plane of incidence and on the direction perpendicular to the plane of incidence, respectively. The prime in $r_{s}^{\prime }$ and $r_{p}^{\prime }$ refers to the fact that the scattered radiation is slightly shifted in frequency and thus also the Fresnel reflection coefficients may be slightly shifted. Combination of Equations (8), (39) and (43) gives:

(44) $$I_{vv}\;\sim \;{\left|\alpha_{xx}\left(1+r_{s}\right)\left(1+r_{s}^{\prime }\right)\right|}^{2},$$

(45) $$I_{hv}\;\sim \;{\left|\alpha_{xy}\left(1+r_{s}^{\prime }\right)\left(1-r_{p}\right)\cos\vartheta +\alpha_{xz}\left(1+r_{s}^{\prime }\right)\left(1+r_{p}\right)\sin\vartheta \right|}^{2},$$

(46) $$I_{vh}\;\sim \;{\left|\alpha_{yx}\left(1+r_{s}\right)\left(r_{p}^{\prime }-1\right)\cos\vartheta {}^{\prime }+\alpha_{zx}\left(1+r_{s}\right)\left(1+r_{p}^{\prime }\right)\sin\vartheta {}^{\prime }\right|}^{2},$$

(47) $$\begin{array}{l} I_{hh}\;\sim \;|\alpha_{yy}\left(1-r_{p}\right)\left(r_{p}^{\prime }-1\right)\cos\vartheta \cos\vartheta^{\prime }\\ \;+\alpha_{yz}\left(1+r_{p}\right)\left(r_{p}^{\prime }-1\right)\sin\vartheta \cos\vartheta^{\prime }+\alpha_{zy}\left(1-r_{p}\right)\left(1+r_{p}^{\prime }\right)\sin\vartheta^{\prime }\cos\vartheta \\ \;+\alpha_{zz}\left(1+r_{p}\right)\left(1+r_{p}^{\prime }\right)\sin\vartheta \sin\vartheta^{\prime }{|}^{2}. \end{array}$$

Equations (44)–(47) are known as the surface selection rules [34,35].

## 5. Polarization Effects for Molecules on Plasmonic Nanostructures

Polarization-dependent effects for molecules located in presence of plasmonic nanostructures are much more complex in comparison to “free” molecules. The principal reason is that the enhancement provided by plasmonic nanostructures is polarization-sensitive and is therefore commonly accompanied by dramatic alteration of polarizations of both the incident beam as well as of the scattered beam. Local field polarization is dictated mainly by coupling of the given light polarization in the metallic nanostructures and the similar process applies in the case of the scattered field. As a result, polarization felt by the molecule can be drastically different from polarization of the incident beam. It causes the depolarization ratio to no longer depend only on the intrinsic properties of the Raman tensor of the analyte, but also (and often mainly) on the geometry of the given nanostructure [39,40,41]. This difference occurs even in the simplest nanostructure geometries, such as a small sphere in the dipole approximation, it can be commonly observed in arrays of elongated nanoparticles (NPs) and is most pronounced for molecules in the location of hot-spots which exhibit anisotropic behaviour [40,42]. Since different polarizations couple to metallic nanostructures with different efficiency, the enhancement factor (EF) can no longer be regarded as a scalar but it takes the form of a tensor. In this case, the polarization and angular dependence of the SERS signal is therefore mostly dictated by the coupling of the laser to the plasmons while symmetry of the Raman tensor of the analyte often plays a minor role [39,40,43].

### 5.1. Polarization Properties of Isolated Spherical Particles

The case of an isolated metallic sphere in the dipole approximation was theoretically studied in [36,44]. Theoretical analysis reveals that the enhanced local field in the vicinity of a small metallic sphere after illumination with a light wave may be expressed in the electrostatic approximation in spherical coordinates as:

(48) $$\vec{E}=E_{0}\left(1+2g\left(\omega \right)\right)\cos\vartheta {\vec{e}}_{r}-E_{0}\left(1-g\left(\omega \right)\right)\sin\vartheta {\vec{e}}_{\vartheta },$$

i.e., the normal field components with respect to the sphere undergo the enhancement by the factor of $A_{r}=1+2g\left(\omega \right)$ while the tangential component is enhanced by the factor of $A_{\vartheta }=1-g\left(\omega \right)$, where $g\left(\omega \right)=\frac{\varepsilon \left(\omega \right)-\varepsilon_{r}}{\varepsilon \left(\omega \right)+2\varepsilon_{r}}$ is the geometrical factor for a sphere. The relative permittivity of the surrounding medium is denoted as $\varepsilon_{r}$. Therefore, amplification of the surface field for any point on the surface of the sphere is determined by the matrix:

(49) $${\overset{↔}{A}}_{r,\vartheta ,\varphi }=\left(\begin{array}{ccc} 1+2g\left(\omega \right) & 0 & 0\\ 0 & 1-g\left(\omega \right) & 0\\ 0 & 0 & 1-g\left(\omega \right) \end{array}\right).$$

As a consequence, molecules with certain elements of symmetry and preferential orientation with respect to the surface will exhibit differences in the relative enhancements of modes belonging to different symmetry classes. Averaging over all solid angles (all positions on the metallic sphere) reveals that the ratio of tangential to normal intensities is:

(50) $$\frac{E_{r}^{2}}{E_{\vartheta }^{2}}={\left|\frac{1+2g\left(\omega \right)}{1-g\left(\omega \right)}\right|}^{2}\frac{\int \cos^{2}\vartheta d\Omega }{\int \sin^{2}\vartheta d\Omega }=\frac{1}{2}{\left|\frac{1+2g\left(\omega \right)}{1-g\left(\omega \right)}\right|}^{2}.$$

In the early 1980s, Moskovits pointed out the existence of three classes of vibrational modes with distinct spectral behaviour (unique excitation profile) [35,45]: (1) those excited only by the normal component of the field and resulting in an induced dipole with a strong component only in the direction perpendicular to the surface; (2) those excited only by the tangential component of the field and resulting in an induced dipole with a strong component tangential to the surface; and (3) mixed cases. EF for respective molecular vibrational modes will thus depend on the orientation of the molecule on the surface. Let us assume that a molecule possessing certain symmetry is adsorbed on the surface so that its principal axis (z axis) is perpendicular to the sphere. Then, the modes of the first type are modes spanning the same irreducible representation as $\alpha_{zz}$. SERS intensity of these modes is expected to obey the relationship:

(51) $$I_{nn}\;\sim \;{\left|1+2g\left(\omega_{i}\right)\right|}^{2}{\left|1+2g\left(\omega_{s}\right)\right|}^{2}.$$

By contrast, modes spanning the same irreducible representation as $\alpha_{xx}$, $\alpha_{yy}$ or $\alpha_{xy}$ benefit only from the existence of the electric field tangential to the surface. Thus,

(52) $$I_{tt}\;\sim \;4{\left|1-g\left(\omega_{i}\right)\right|}^{2}{\left|1-g\left(\omega_{s}\right)\right|}^{2}.$$

Finally, modes excited by the normal component of the field and resulting in an induced dipole with a strong component only in a direction parallel to the surface, or vice versa, will be characterized by the excitation profile:

(53) $$I_{nt,tn}\;\sim \;\frac{1}{2}({\left|1+2g\left(\omega_{i}\right)\right|}^{2}{\left|1-g\left(\omega_{s}\right)\right|}^{2}+{\left|1+2g\left(\omega_{s}\right)\right|}^{2}{\left|1-g\left(\omega_{i}\right)\right|}^{2}).$$

**Figure 7:**
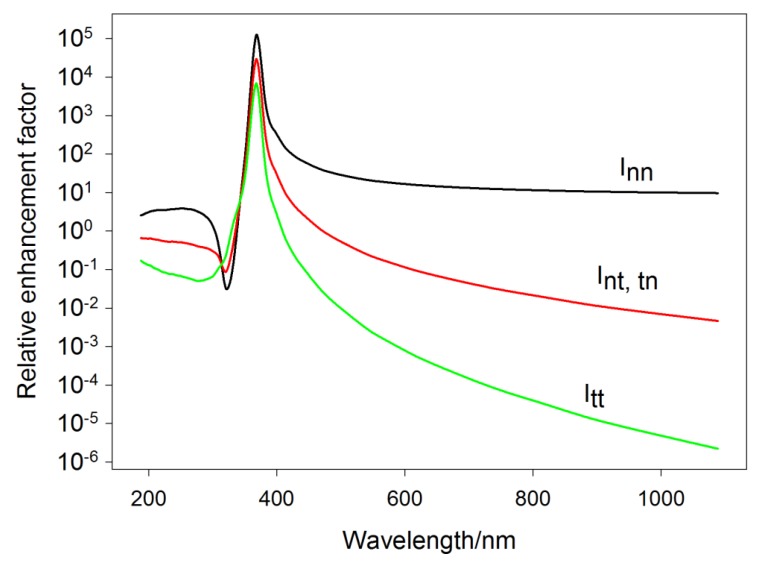
Calculated wavelength-dependent relative enhancement factor for molecules adsorbed in the vicinity of a small silver sphere for the three classes of Raman modes. $\varepsilon_{r}$ was assumed to be 1.33.

As usual, $\omega_{i}$ denotes frequency of the excitation radiation and $\omega_{s}$ frequency of the scattered radiation. It is clear that the SERS excitation profiles associated with the three types of modes obey the wavelength dependence of the right-hand sides of Equations (51)–(53) (Figure 7). By contrast, when the molecule adsorbs with the z axis tangential to the surface, the role of z and x (or y) in the character table is to be swapped. This fact provides a unique tool for determination of molecular orientation on nanostructured metallic surfaces [45,46,47,48].

Due to disrupted spherical symmetry in the case of non-spherical particles, the polarization insensitive SERS EF is expected to split into two (or more) geometry-dependent components. For example, in the case of prolate spheroids, only the incident field polarized parallel to the long axis results in the greatest surface fields [49]. Such electrodynamic calculations were performed already in the 1980s and their understanding is a key for elucidation of the polarization-resolved optical response of more complex systems such as elongated particles or nanorods (NRs), which will be discussed in more detail in the second part of this section.

### 5.2. Local Field Distribution in Presence of Hot-Spots and Its Relation to Molecular Orientation

Unfortunately, the optical response of very few plasmonic systems can be computed as straightforwardly as shown in Section 5.1. Both the experimental results as well as the theoretical calculations show that electromagnetic field can be confined in nanometer-sized metallic clefts. Analytical treatment of a two sphere system, performed already in the 1980s [50,51], suggests that molecules residing in a nanogap between the NPs are subjected to ~10^5^× stronger electromagnetic field in comparison to the field enhanced by one single NP. Due to mutual interaction, electromagnetic field in such cavities is much larger than the sum of the fields caused by two non-interacting spheres, which has been explained by strong electromagnetic coupling between the NPs, giving rise to coupled plasmon resonances [13,35,52,53,54]. Coupled plasmon resonances are characterized by much dramatic difference in responses to different polarizations. Such an extreme enhancement occurs only if the incident polarization is along the interparticle axis. For light polarized across the interparticle axis, the enhancement is almost negligibly different from its value at a single, isolated particle [40,42]. Closer analysis shows that with NP radii of 45 nm (average size of the silver NPs used) and the separation distance of d= 5.5 nm (diameter of a hemoglobin molecule, [52]), the enhancement effect adds another ~2–3 orders of magnitude in comparison to isolated NPs and further ~2–3 orders of magnitude after decreasing the NP gap down to ~1 nm, making the total EF of ~10^11^. Nowadays, SERS EFs of ~10^11^–10^12^ are considered to be theoretical limits (for silver) on account of the electromagnetic contribution, sufficient even for observation of single-molecular SERS [55,56,57]. Not surprisingly, there often exists an inverse relationship between the EF and reproducibility of the substrate used (“SERS uncertainty principle”).

The extremely spatially-localized sites providing extreme enhancement were dubbed hot-spots in the literature [13]. However, geometry of hot-spots is not restricted only to two mutually interacting spheres. Generally, for systems of aggregated particles with nanoscale crevices or junctions where interparticle separation can be made very small, extremely localized regions of ultrahigh enhancement were predicted [53] as well as experimentally demonstrated [56,58,59,60]. It means that the enhancement in the gap region where majority of the electromagnetic energy is packed completely overwhelms the surface average and produces the dominant contribution to single-molecular sensitivity in SERS. Besides, theory also predicts very strong enhancement of the electromagnetic field at sharp metallic tips and large curvature regions due to so-called lightning-rod effect. In hot-spot dominated systems, information on the symmetry of the Raman tensor (or molecular depolarization ratio, Equation (24)) is almost completely overridden by anisotropic pattern of the nanostructures. 

**Figure 8:**
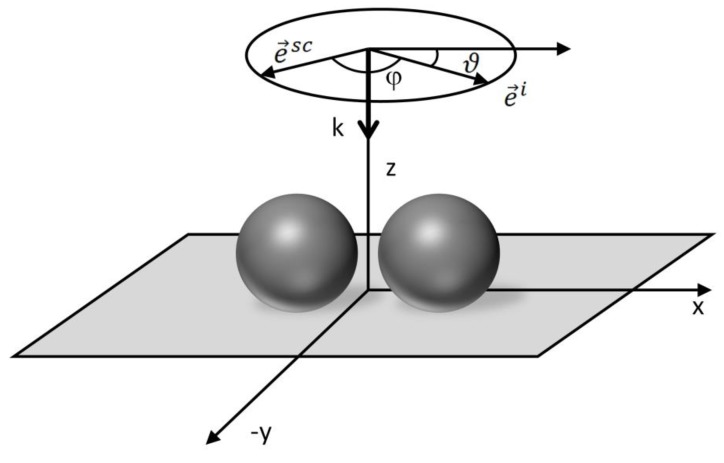
Geometry of a dimer. The Cartesian coordinate system is chosen so that the dimer axis lies in the x direction, the excitation beam travells along the z direction with the incident polarization making an angle ϑ with the x axis. Scattered radiation is assumed to be collected in a backscattering geometry, allowing only the polarization making an angle φ with respect to the incident polarization (i.e., making an angle ϑ+φ with respect to the x axis) to be detected.

**Figure 9:**
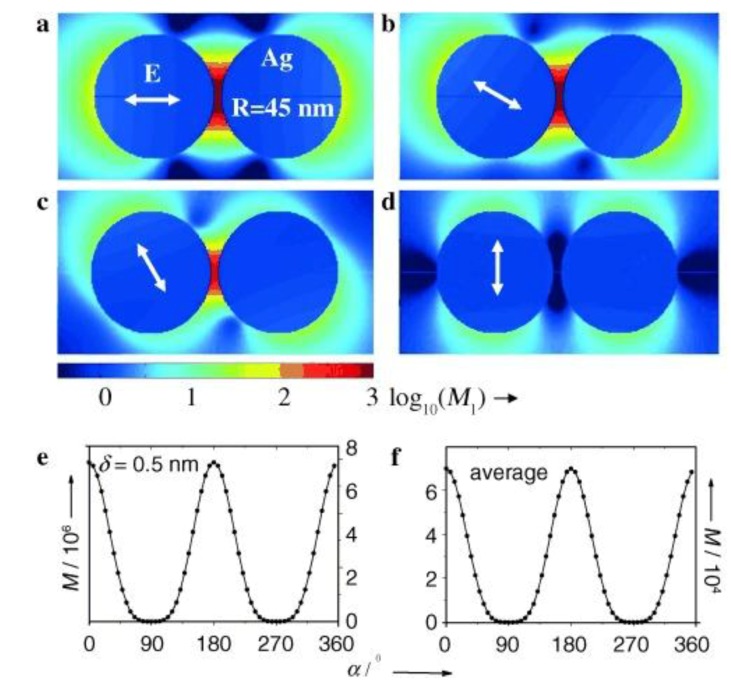
Local intensity enhancement $M_{1}={\left(\frac{E_{loc}}{E_{0}}\right)}^{2}$ in a logarithmic scale in a plane through the centers of the Ag spheres and perpendicular to the incident wavevector $\vec{k}$ versus incident polarization: (**a**) 0°; (**b**) 30°; (**c**) 60° and (**d**) 90°. The incident wavelength was 514.5 nm in all cases. The arrows represent the different polarizations. In (**e**), the SERS enhancement factor $M=M_{1}^{2}$ is shown as a function of the incident polarization α for a point in the nanogap located at the dimer axis δ=0.5 nm away from one spherical surface, and the fit (solid line) to a $\cos^{4}\alpha$ dependence. In (**f**), M averaged over all points δ=0.5 nm outside the Ag sphere surface versus α and fit to a $\cos^{4}\alpha$ dependency are shown. The radius R=45 nm corresponds to the average size of the Ag nanoparticles used in the experiment while the separation distance d=5.5 nm corresponds to the diameter of a hemoglobin molecule. Reprinted with permission from [54].

Let us take a closer look at the simplest case of a hot-spot—a dimer formed by two metallic NPs. Let us adopt such a Cartesian system of coordinates that the dimer axis is parallel with the x axis as depicted in Figure 8. In this case, the optical response of a molecule situated in the gap between the two NPs is completely dominated by polarization in the x direction (Figure 9). 

As already mentioned, the ratio of obtained intensities for polarization parallel/perpendicular to the dimer axis may amount to 10^5^. Therefore, the SERS response for polarization perpendicular to the dimer axis can be neglected (the enhancement effect of the dimer is comparable to that of one isolated particle in this case). Thus, assuming that the incident wavevector lies in the z direction, local field strength for polarization making an angle ϑ with respect the x-axis will be:

(54) $$E_{local}=\gamma E_{0}\cos\vartheta .$$

In other words, amplification of the surface field is of tensorial nature and can be expressed as:

(55) $$\overset{↔}{A}=\left(\begin{array}{ccc} \gamma & 0 & 0\\ 0 & 1 & 0\\ 0 & 0 & 1 \end{array}\right).$$

The factor γ represents amplification of the laser field polarized along the x-axis. Similarly to the incident radiation, also the scattered radiation exhibits different coupling efficiency depending on the angle made between the dimer axis. Thus, the local field should scale as ~cosϑ and the local intensity as $\sim \cos^{2}\vartheta$. Assuming the backscattering geometry and for simplicity the same EF for both the incident and the scattered radiation, respective intensities obey the following trends:

(56) $$I\left(180{}^{\circ},{⏊}^{sc},{⏊}^{i}\right)=I\left(180{}^{\circ},{\|}^{sc},{\|}^{i}\right)\;\sim \;\cos^{4}\vartheta ,\;(\mathrm{parallel}\;\mathrm{polarizations}),$$

(57) $$I\left(180{}^{\circ},{\|}^{sc},{⏊}^{i}\right)=I\left(180{}^{\circ},{⏊}^{sc},{\|}^{i}\right)\;\sim \;\cos^{2}\vartheta \sin^{2}\vartheta ,\;(\mathrm{crossed}\;\mathrm{polarizations}),$$

and the depolarization ratio is:

(58) $$\rho ={\mathrm{tg}}^{2}\vartheta ,$$

which diverges for ϑ=90°. In this crudest approximation, the depolarization ratio is determined solely by the plasmonic nanostructures and is not by any way linked to the molecular depolarization ratio.

The theoretical predictions above may be easily verified experimentally in the case of colloids. Colloids may be regarded as a collection of randomly oriented hot-spots where the overall SERS signal usually originates from a few molecules residing in the hot-spot sites and the rest occupying the less enhancing sites [40]. Although an extremely small percentage (difficult to determine precisely) of molecules actually cover the hottest sites, their presence is decisive for the total enhancement [61,62]. Integration over random orientations of a collection of dimers in standard azimuthal coordinates (Equation (5)) yields:

(59) $$\rho =\frac{\iint \cos^{2}\vartheta \sin^{2}\vartheta \cos^{2}\varphi d\Omega }{\iint \cos^{4}\vartheta \;d\Omega }=\frac{1}{3},$$

which is the expected value of the depolarization ratio observed experimentally [41,43]. Extremely low detection limits for molecules adsorbed on hot-spot dominated SERS active systems opposed to almost complete loss of more detailed information on the symmetry of the vibrations involved may be viewed as another consequence of the SERS uncertainty principle. 

**Figure 10:**
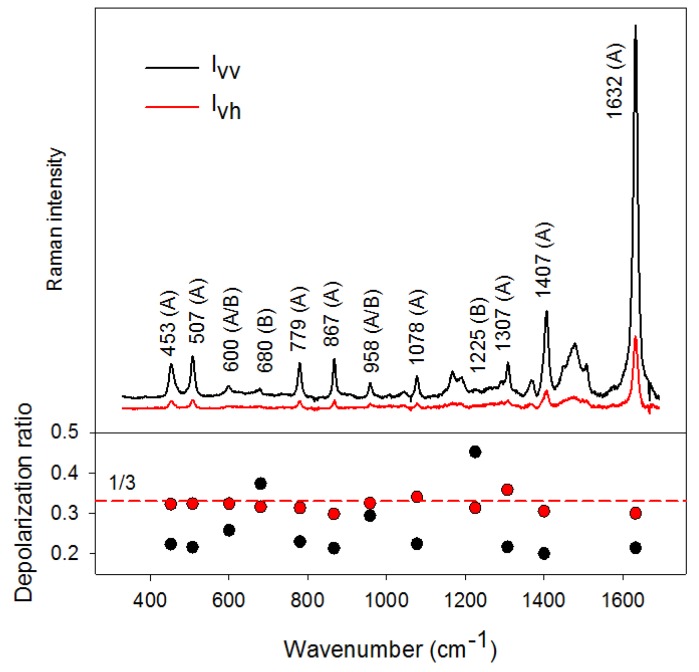
Polarized (preresonance) Raman spectra of methylene blue (MB) in a water solution (excitation wavelength 532 nm; after fluorescence baseline subtraction) and corresponding depolarization ratios (black dots). Symmetry species of respective vibrational modes are indicated in the upper part. Comparison with the depolarization ratios obtained from hydroxylamine-reduced Ag NPs is indicated by red dots (SERS spectra are not shown for better clarity). MB concentration was 10^−4^ M, laser power 100 mW, accumulation time 5 min.

Let us demonstrate some of the previous theoretical aspects on the Raman spectra of methylene blue (MB) measured in our laboratory in both $\left(90{}^{\circ},{⏊}^{s},{⏊}^{i}\right)$ ($I_{vv})$ and $\left(90{}^{\circ},{\|}^{s},{⏊}^{i}\right)$ $\left(I_{vh}\right)$ experimental configurations (Figure 10). MB was in the form of a water solution (concentration 10^−4^ M) and in hydroxylamine-reduced Ag NPs (final MB concentration of 10^−6^ M), prepared by the standard procedure [63]. In both cases the excitation wavelength was 532 nm, therefore both spectra should be considered as preresonance ones. This brings certain difficulty with fluorescence and the need for baseline subtraction in the case of non-SERS measurements, which makes determination of respective peak intensities more difficult, however, all basic principles highlighting the differences in Raman/SERS depolarization ratios are retained.

MB belongs to the $C_{2}$ point group possessing 108 normal vibrational modes distributed among A and B symmetry species as $\Gamma^{3N-6}=54A⊕54B$ (A denoting the totally-symmetric vibrations and B the non-totally symmetric vibrations). Respective bands in Figure 10 are labelled as A or B on the basis of theoretical calculations adapted from [64].

From Figure 10, several conclusions can be drawn. First, it is obvious that the most intense bands in preresonance Raman (non-SERS) spectrum are almost entirely the A species, which is fully consistent with the theory of resonance Raman scattering. Second, evaluation of the Raman depolarization ratios reveals that for the A species ρ lies between ~0.19 and 0.23 while for the B species ρ is up to ~0.45, approximately twice as much as for A. This is again in full compliance with the theory according to which the depolarization ratio of symmetric vibrations (A) is always lower than for non-totally symmetric vibrations (B). However, this difference in the depolarization ratios between A and B species is wiped out completely when the measurement is performed on a silver colloid where the depolarization ratios are (within the experimental error) all around 1/3, irrespective of the symmetry of the vibration involved. Since at lower analyte concentrations, molecules are expected to adopt sub-monomolecular surface coverage and tend stack up in multiple layers at higher analyte concentrations, series of concentration-dependent polarization-resolved SERS measurements while monitoring the depolarization ratios could represent a unique way to distinguish these two regimes.

**Figure 11:**
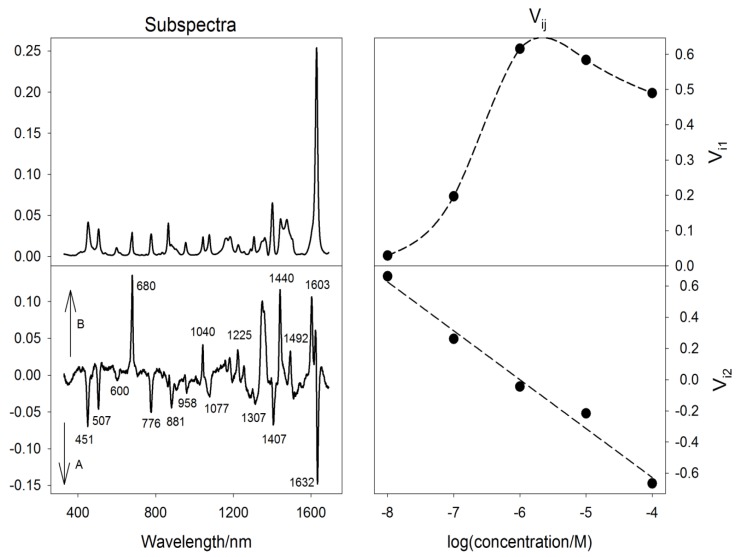
Results of factor analysis of concentration-dependent SERS measurements of methylene blue (MB). Profile of the V_i1_ coefficients reflects the variation in MB SERS intensity with concentration. Profile of the V_i2_ coefficients and the shape of the second subspectrum reveal a systematic decrease in the ratio of EFs for B/A modes with increasing concentration (cf. Figure 10).

Figure 11 shows our results from concentration-dependent MB SERS measurements. Singular value decomposition algorithm [65] based on factor analysis was employed to treat the spectral set.

Profile of the V_i1_ coefficients reflects the variation in total MB intensity with concentration. As expected, Raman intensity rises when MB concentration is increased from 10^−8^ M to ~ 10^−6^ M concentration but starts to decrease when further increasing MB concentration, probably due to MB aggregation and less efficiency in occupying the highest-enhancing sites. After normalizing the original spectral set using the V_i1_ coefficients, the V_i2_ coefficients were found almost perfectly linear (in a semi-logarithmic scale) with concentration with the second subspectrum highlighting the differences between A and B symmetry modes. In other words, when increasing MB concentration, the ratio of EFs for B/A modes is systematically decreasing. This finding clearly reveals a change in MB adsorption geometry with concentration, adopting a face-on adsorptive stance at lower concentrations and a preferable edge-on adsorptive stance at higher concentrations [64,66].

Efforts to make use of the enhancement capability of dimers are still very lively in literature, which can be documented by numerous studies from the last years [67,68,69,70,71,72,73,74], benefiting from extremely high enhancement between two adjacent metallic NPs or between a metallic NP and a substrate. A very promising analytical tool for biosensing applications include DNA origami, [75,76,77] where NPs with defined distance can be constructed. However, all these illustrations raise a question about whether or not there exists a general rule to reveal some information regarding the Raman tensor elements of the analyte (intrinsic properties of the SERS probes) by measurements on nanostructured metallic surfaces. Correlation between the morphological anisotropy and symmetry of the vibrational species was found in [78]. Using a well-established pyridine molecule, the authors demonstrated that the in-plane (ring stretching) vibrational modes are sensitive to the morphological anisotropy while the out-of-plane (ring deformation) modes are not. A more in-depth approach was developed by P. Gucciardi et al. [31,39,43], who in his analysis went beyond the commonly used $E^{4}$ approximation. For a hot-spot represented by a dimer with the axis lying in the x direction, the authors used the field enhancement tensor in the form:

(60) $$\overset{↔}{A}\left(\omega_{i}\right)=\left(\begin{array}{ccc} \gamma & 0 & 0\\ 0 & 1 & 0\\ 0 & 0 & 1 \end{array}\right),$$

and the re-radiation enhancement tensor as:

(61) $$\overset{↔}{A}\left(\omega_{sc}\right)=\left(\begin{array}{ccc} \gamma \left(1+\varepsilon \right) & 0 & 0\\ 0 & 1 & 0\\ 0 & 0 & 1 \end{array}\right),$$

where ε is a small perturbation accounting for the difference between the enhancement for the incident and the scattered radiation. The local field felt by the analyte and the amplified scattered field are thus:

(62) $${\vec{E}}_{local}=\overset{↔}{A}\left(\omega_{i}\right){\vec{E}}_{i},\;\;{\vec{E}}_{SERS}=\overset{↔}{A}\left(\omega_{sc}\right){\vec{E}}_{sc}.$$

These two fields are actually those that enter Equation (8). The theoretical SERS intensity for many randomly oriented molecules as a function of the angle ϑ and φ (see Figure 8) was found to be:

(63) $$\begin{array}{c} I\;\sim \;\gamma^{4}{\left(1+\varepsilon \right)}^{2}\langle \alpha_{ii}^{2}\rangle \cos^{2}\vartheta \cos^{2}\left(\vartheta +\varphi \right)+\gamma^{2}\left(1+\varepsilon \right)\langle \alpha_{ii}^{2}\rangle \left[1/2\sin2\vartheta \sin2\left(\vartheta +\varphi \right)\right]\\ +\gamma^{2}\langle \alpha_{ij}^{2}\rangle {\left[\varepsilon \sin\vartheta \cos\left(\vartheta +\varphi \right)+\sin\varphi \right]}^{2}+\langle \alpha_{ii}^{2}\rangle \sin^{2}\vartheta \sin^{2}\left(\vartheta +\varphi \right). \end{array}$$

Equation (63) indicates that the total intensity contains a term proportional to $\gamma^{4}$ ($E^{4}$ approximation) but also other terms beyond the $E^{4}$ approximation. More importantly, this equation also suggests how the molecular depolarization ratio (the quantity corresponding to $\langle \alpha_{ij}^{2}\rangle /\langle \alpha_{ii}^{2}\rangle$ that would be measured for a “free” molecule without the presence of metallic nanostructure) is influenced by the EF and the experimental geometry. The diagonal terms of the Raman tensor of the analyte are enhanced as $\sim \gamma^{4}$ but the non-diagonal terms only as $\sim \gamma^{2}$, which suggests that with higher γ, the effect of the geometrical shape of the nanostructure will be dominant. Since the role of the EF γ in Equation (63) is crucial, virtually causing linearization of the polarization of both the incoming and the outgoing light, it remains very difficult to verify this formula experimentally [31,79]. More detailed discussion can be found in [31].

### 5.3. Polarization Properties of Trimers and Aggregated NP Domains

**Figure 12:**
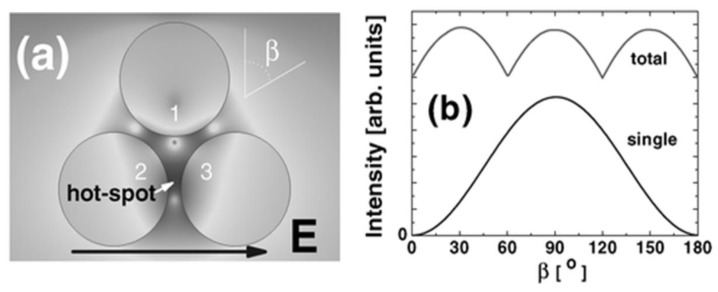
A simple example of a cluster with quasi-isotropy. In this case an example of a 2D cluster formed by three cylinders (radii = 25 nm) with a small separation gap (d = 6.7 nm) is shown. The problem is solved in the electrostatic approximation, wavelength = 357 nm. The polarization is changed in the plane for different angles β as shown in (**a**) where the electromagnetic intensity (on a logarithmic grey-scale) is explicitly shown for the case β=90°. A hot-spot in between cylinders 2 and 3 can be easily seen. If we look at the maximum enhancement at any point on the surface of the cluster, there will always be a place that profits the most from the particular orientation of the field and we obtain the curve labelled as ‘‘total’’ in (**b**). The three maxima in the latter are the three possible two-cylinder hot-spots in this cluster that achieve their highest value when the field is aligned along the axis joining any two of them. If the surfaces were uniformly covered with analyte, an almost isotropic response is obtained. The degree of isotropy increases with the complexity of the cluster. However, if the enhancement at one specific point is monitored (in this case the enhancement in the gap in between 2 and 3) we obtain the curve labelled as ‘‘single’’ in (**b**) which shows the full anisotropy of any single hot-spot. Adapted from [40] with permission from The Royal Society of Chemistry.

Polarization properties of trimers have been studied in [80]. The third particle breaks the dipolar symmetry of the two-particle junction, generating a wavelength-dependent polarization pattern, which was not observed in the case of a dimer. In general, polarization-dependent profile of more complex NP clusters is expected to exhibit multiple maxima and minima, often reflecting symmetry of the nanocluster array [40] (Figure 12). If the enhancement at one specific point were monitored (such as a gap between 2 adjacent NPs), full anisotropy would be observed. Although the SERS characteristics of any single probe-adsorption were found to depend on polarization for trimers in [81] as well as supported by finite-difference time-domain (FDTD) simulations, the periodic array of regular Ag NP trimers was found to form a polarization-independent SERS signal. This fact was attributed to a global structural D_3h_ symmetry of the nanopit area. The polarization independence was even better as compared to large-area Ag nanowires and randomly disordered Ag NPs of uneven size, producing clearly discrepant SERS signals under different incident polarizations. Thus, supposing the surface is uniformly covered with an analyte, such clusters will show a considerable degree of isotropy as a whole [58,82,83]. 

Similar conclusion was supported in [84] using arrays of NRs prepared by inkjet printing technique, or by Shegai in [85]. Shegai attributed different depolarization ratios of different rhodamine 6G bands adsorbed on silver nanocrystal aggregates to charge transfer resonances since the electromagnetic field around the nanocrystals was found near-isotropic. A more theoretical approach was adopted in [86]. Making use of the group theory, the authors showed that a nanostructure that belongs to *C_n_* symmetry point group (n≥3) has an optical response that is insensitive to the light polarization when the wavevector is parallel to the *C_n_* axis. Attempts have been made to verify this claim experimentally using arrays of nanotriangles, nanostars and other nanoobjects [87]. However, due to non-negligible relative standard deviation of the SERS measurements and the fact that the theoretical criteria mentioned above are not perfectly met, the decision whether or not there exists a polarization-dependent pattern is often difficult.

### 5.4. Polarization Properties of Regular Solid Plasmonic Platforms

**Figure 13:**
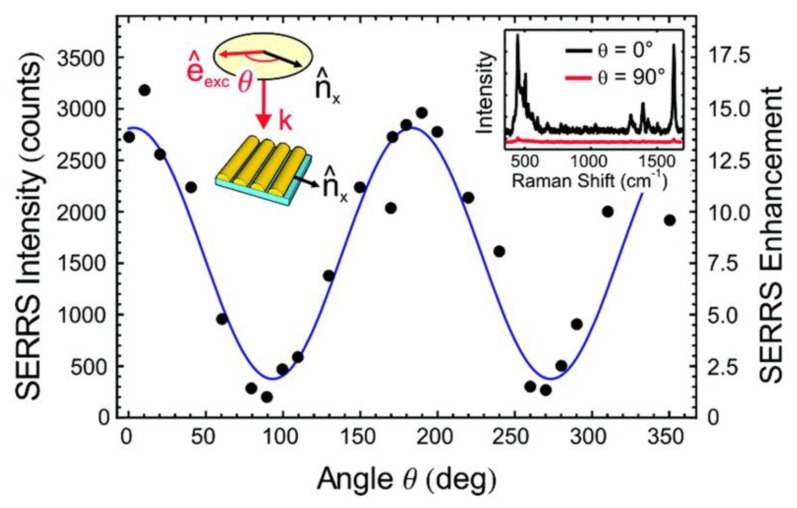
Unpolarized SERS intensity of methylene blue adsorbed on gold nanowires (NWs) versus excitation polarization θ as measured in a backscattering geometry. SERS intensity was found maximum for θ=0° (inset, black line), i.e., for incident polarization vector (denoted as ${\vec{e}}_{exc}$) parallel to the nanocavity axis ${\vec{n}}_{x}$, and minimum for θ=90° (inset, red line), i.e., ${\vec{e}}_{exc}$ parallel to the NWs long axis ${\vec{n}}_{y}$. The SERS intensity profile was well fitted with the $\sim \cos^{2}\vartheta$ law (blue line), plus low order terms (Equation (63)). Excitation wavelength was 633 nm, thus the SERS spectrum is considered as a resonance one (SERRS). Adapted with permission from [43]. Copyright 2011 American Chemical Society.

**Figure 14:**
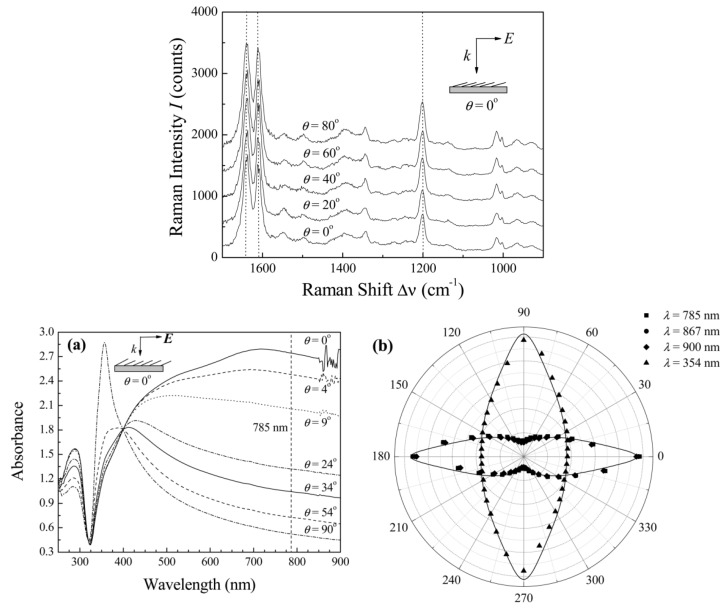
(**a**): Polarized SERS spectra of trans-1,2-bis(4-pyridyl)-ethene (BPE) on Ag nanorod substrates as a function of the incoming polarization angle measured in a backscattering geometry. (**b**): Polarized UV/Vis/NIR absorption spectra. Cross-section of the NR array is schematically depicted in the inset (inclination angle of the nanorods was ~ 71° with respect to the surface normal). Both diagrams show the p-polarization direction with the incident polarization almost parallel to the major long axis of the nanorods. The maximum SERS intensity was observed in the polarization direction perpendicular to the long axis of the Ag nanorods, while the UV/Vis absorbance was strongly polarized along the direction of the long axis of the NR array. Adapted with permission from [97]. Copyright 2006 American Chemical Society.

**Figure 15:**
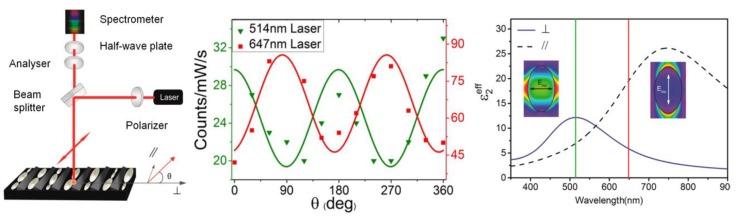
**Left:** Setup for Raman measurements used. Middle: Normalized intensity of the 1079 cm^−1^ band of 4-mercaptobenzonitrile (MBN) adsorbed on anisotropic Ag nanoparticle arrays created by metal evaporation on rippled silicon substrates as a function of θ obtained with the 514 nm (green triangles) and the 647 nm (red squares) excitation. The respective solid lines correspond to fits of a $\cos^{2}\theta$ (green) and $\sin^{2}\theta$ (red) function to the data. **Right:** Imaginary part of the effective dielectric function of the Ag film parallel and perpendicular to the long axis of the ripples. Adapted with permission from [116]. Copyright 2016 American Chemical Society.

Considerations above can be analogously extended to many other plasmonic platforms possessing anisotropic morphology fabricated using various procedures [88]. For example, polarization properties of metallic nanocubes [89,90,91], arrays of silver NP rows [92], gold nanoassemblies [93], metallic gratings structures [94], half-shells [95] etc. were studied in literature. A frequently-studied SERS-active system from the point of view of polarization and angular characteristics are arrays of elongated NPs appearing under different names in literature such as NRs [96,97,98,99], nanowires [43,60,100,101,102,103], nanoantennas [104,105], nanorattles [106,107], nanobones [108] or NP-nanowire systems [109,110,111] where the ~cosϑ trends were often observed (Figure 13, Figure 14 and Figure 15). Published results indicate that the optical response for light polarized parallel/perpendicular to the long axis of the nanoobjects (related to excitation of longitudinal plasmon modes (LM) or transverse plasmon modes (TM), respectively) is indeed different. However, the SERS intensity profile with varying angle/polarization is a function of a wide range of parameters, such as the dimensions of the metallic objects, their aspect ratios and spatial arrangement, material (Ag or Au), the excitation wavelength or orientation of the probe molecules on the surface [85,112,113], which resulted in seemingly contradictory accounts appearing in literature. Last but not least, what hampers more precise analyses of the angular and polarization effects is sample bleaching and photodecomposition induced by the incident laser, which causes poorer reproducibility and brings further uncertainty to the set of experimentally measured points. This aspect was usually resolved in the literature by applying a correction assuming exponential decay of the SERS signal with time [81,97,109]. Polarization-dependent properties of SERS microarrays also found use in elimination of the polarization insensitive spurious bands originating from the bulk material [94].

Although theory predicts the difference in the EFs for different light polarizations up to ~10^5^ (for an ideal structure [53,54]), in reality, this factor is much more modest. For example, the ratio of SERS responses in the directions parallel/perpendicular to the NRs was found to be around 0.8 for silver NRs as reported in [97]. This finding was attributed to the lateral arrangement of the NR lattice and strong electromagnetic coupling between adjacent metallic NRs instead of preferential molecular orientation of the probe molecule on the surface. A similar trend was observed for coupled metallic nanowires [60], oriented silver nanowire films [101], silver nanowire rafts [100] or self-organized gold nanowires [43]. In all these cases the SERS intensity ratios for light polarized perpendicular/parallel with respect to the NR axes was found around 5 (see Figure 13) and explained by excitation of a new plasmon mode trapped in the interstices between adjacent, parallel wires, similarly to the case of a dimer. However, this only happens if the interwire distance is sufficiently small (<~10 nm) [60]. By contrast, antenna arrays similar to bent needles [104], nanorattles [106], single gold NRs [99] or aligned Ag NRs prepared by a dynamic oblique deposition technique [98] were all found to exhibit the enhancement higher for excitation light polarized parallel to the needle/NR direction than for the perpendicular case. Such results were, on the other hand, usually rationalized by intense local electromagnetic fields emanating from points of high curvature, such as NR tips (“lightning-rod effect”) and the dominant role of the longitudinal plasmon modes. To sum up, the experimentally measured polarization dependences often may be understood as a result of “competition” between the enhancement provided by longitudinal plasmon modes and by transverse plasmon modes. For example, for NRs growing on a substrate in one specific direction as indicated in Figure 14, the obtained SERS intensities are expected to be:

(64) $$I\;\sim \left[a_{\left|\right|}{\left({\vec{e}}^{i}\cdot \vec{l}\right)}^{2}+a_{⏊}\left(1-{\left({\vec{e}}^{i}\cdot \vec{l}\right)}^{2}\right)\right]\cdot \left[a_{\left|\right|}{}^{\prime }{\left({\vec{e}}^{sc}\cdot \vec{l}\right)}^{2}+a_{⏊}{}^{\prime }\left(1-{\left({\vec{e}}^{sc}\cdot \vec{l}\right)}^{2}\right)\right],$$

where ${\vec{e}}^{i}$ is the unit incident field vector, ${\vec{e}}^{sc}$ is the unit scattered field vector, $\vec{l}$ is the unit vector determined by the long axis of the NRs and $a_{\left|\right|}$ and $a_{⏊}$ are factors determining the plasmonic response to polarization parallel/perpendicular to the long axis of the NRs for the excitation light (unprimed values) and the scattered light (primed values). The first bracket in Equation (64) accounts for the enhancement of the incident radiation and the latter accounts for the enhancement of the scattered radiation. What matters is which of the two factors $a_{⏊}$ and $a_{\left|\right|}$ is larger. In general, the answer to this question is not uniform since it is extremely sensitive to the specific preparation procedure of given plasmonic nanostructures. As a rule of thumb, for arrays of regular NRs stacked in close proximity to each other (gap < ~ 10 nm), the role of factor $a_{⏊}$ usually prevails. Similar conclusion holds true for NP-nanowire systems. On the other hand, for rather isolated or well-separated NRs, the most contributing term to the biggest SERS sensitivity will usually be the factor $a_{\left|\right|}$ [114,115].

Sensitivity of the plasmonic anisotropy to the excitation wavelength was investigated in more detail in [116] where two different excitation wavelengths were used to study the polarization-dependent SERS on anisotropic Ag NP array. The study demonstrates that SERS nanostructures can possess completely different polarization characteristics when using two different wavelengths. The $\cos^{2}\vartheta$ and $\sin^{2}\vartheta$ trends were found using the 514 nm and 647 nm excitation wavelength, respectively (Figure 15). Completely different polarization characteristics were in good correspondence with the dielectric function parallel and perpendicular to the long axis of the nanostructures as determined by spectral ellipsometry. This approach is expected to open novel possibilities in biosensing applications due to increase in the specificity of target detection by using multiple excitation wavelengths. 

### 5.5. Angular Dependences in SERS

As far as angular dependences are concerned, the situation in SERS is again more complicated in comparison to classical Raman measurements (Figure 4) due to the presence of a third element—the nanostructure, which imposes its own anisotropic pattern in the directional properties of the SERS signal. Moreover, the term angular dependences itself is rather ambiguous since “angle” may be understood either as the angle between laser wavevector/polarization vector and a specific nanostructure, or as the angle determining the specific illumination-observation geometry in a way analogous to that described in Section 3. From the point of view of the latter meaning, the vast majority of experiments have been performed only in backscattering geometry, which means that a detailed inspection on the angular dependence of the SERS signal is somewhat exceptional in the literature. However, possible optimization of plasmon-based sensors for maximum signal enhancement relies, among other things, on the right choice of the excitation angle and/or the scattering angle. Geometric reasons, such as change in the size of the illuminated area and possibly different efficiency in collection of the scattered radiation must be also taken into account when aiming to retrieve angular dependences [112,113,117]. This was often neglected. The issue of optimum excitation and collection efficiency is of great importance also in the construction of optical waveguides with the aim to minimize losses where coupling of the SERS field to the waveguide modes must be carefully optimized. This approach is forecasted to open the way towards lab-on-a-chip sensing systems, allowing multiplexed detection of extremely weak Raman signals on a highly dense integrated platform [118,119].

**Figure 16:**
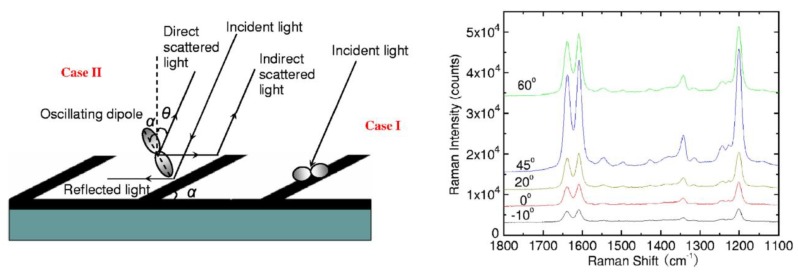
**Left:** Schematic illustration of the modified Greenler’s model of an induced dipole on a Ag nanorod: Case I, where the dipole is perpendicular to the incident plane; Case II, where the dipole is on the incident plane. All the induced dipoles are perpendicular to the NR. **Right**: Representative SERS spectra of trans-1,2-bis(4-pyridyl)-ethene adsorbed on the Ag NR substrate at different incident angles θ. The peak intensity was strongest at the angle θ around 45°, which was in agreement with the model. Adapted with permission from [96].

A basic idea of the simplest models trying to elucidate the SERS angular characteristics again lies in the fact that the primary electric field felt by the molecule is the sum of the incident and the reflected field, which induces an oscillating dipole in the adsorbed molecule. A modified Greenler model [5] based on classical electrodynamic dipole radiation was used to explain the anisotropic nature of tilted Ag NRs, producing maximum SERS intensities at approximately 45° relative to the surface normal [96], but measured still in the backscattering configuration (Figure 16). Although the length of the NRs used (~ 868 nm) was comparable to the excitation wavelength (785 nm) in this case, the model was treating the surface of the NR as planar, neglecting the diffraction effect and calculating near-field intensities using the Fresnel equations. Later, the authors upgraded their model, assuming that the primary field at the NR surface is the sum of the incident and reflected fields from the Ag NR and from the supporting Ag film. The authors showed that the presence of an underlying Ag film plays a crucial role for the SERS enhancement due to its high reflectivity [120]. Although these simple considerations were proven to capture the essential angular characteristics in SERS, NRs of subwavelength dimensions can be misleading to treat as planar as demonstrated in [121]. There, a strong difference between the optical constants of the NR films and those of the constituent materials were found using generalized ellipsometry (Jones formalism). 

Unlike optical constants of homogeneous materials, optical constants of nanostructured layers depend (due to the presence of subwavelength structures) also on the incident angle. For example, island-like Ag films in [122] exhibited uniaxial optical properties and NRs in [121] exhibited biaxial properties with the complex refractive index different for different orientations of the incident angle with respect to the NRs, similarly to the case of crystals [123]. A very similar conclusion was drawn from our work [112] where we showed that the optical constants of silver NR arrays are sensitive to both the incident angle as well as rotation of the substrate by 90°. On the other hand, they were fairly insensitive to flipping the substrate by 180°. Moreover, surface roughness or the presence of plasmonic resonance may invoke a partial depolarization, i.e., a loss of coherence of the phase and amplitude of the electric field [124]. These phenomena lie beyond the validity of the Jones model and their full description requires using more complicated Stokes formalism [125].

**Figure 17:**
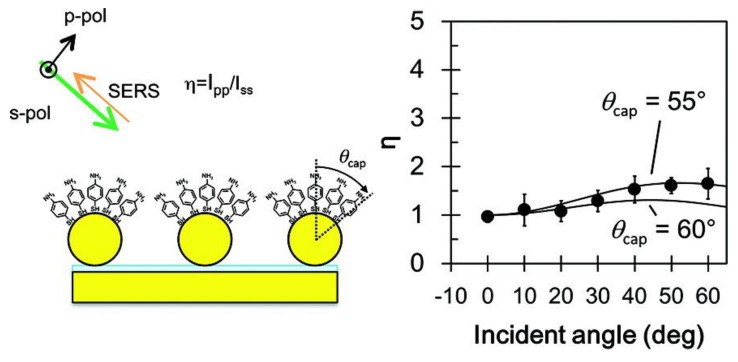
**Left:** Scheme of the angular-resolved polarized SERS of aminobenzenothiol immobilized on gold nanospheres. **Right:** Observed depolarization ratio (η) profile. The solid lines represent different η profiles simulated for different $\theta_{cap}$ angles. Adapted with permission from [113]. Copyright 2012 American Chemical Society.

An important contribution to angular- and polarization-resolved SERS was made in [113], probing adsorbate-covered gold NPs immobilized on a metallic substrate (Figure 17). Mathematical background combining local field enhancement tensor, intrinsic molecular Raman tensors and rotation tensors has been developed in order to elucidate the surface coverage depending on the analyte concentration. The authors also pointed out the role of the surface tension of the solution used, preventing access of the analyte molecules in between the nanospheres in the high concentration regime. 

**Figure 18:**
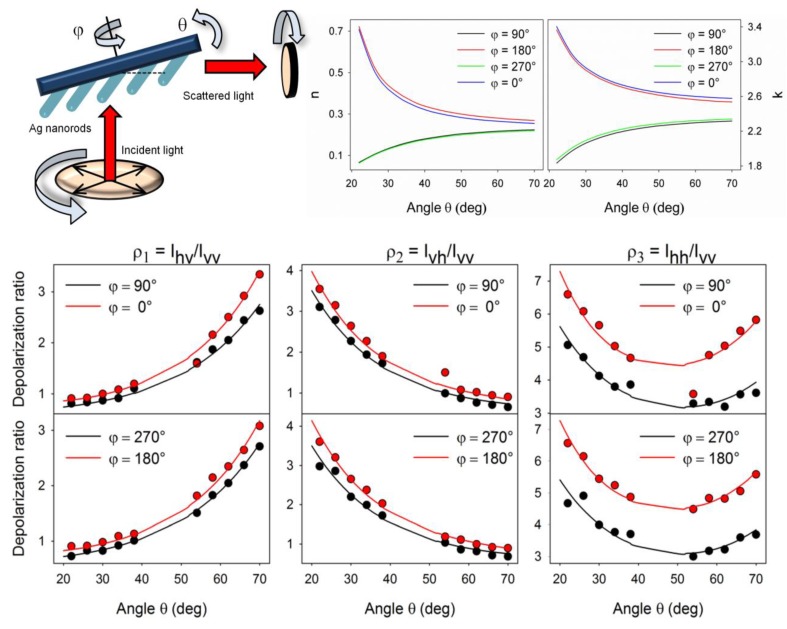
**Upper left:** Scheme of the experimental geometry used for polarization- and angular-resolved SERS on silver nanorod arrays using methylene blue (MB) as the probe molecule. **Lower part:** Depolarization ratio profiles for the 1628-cm^−1^ MB band for different angular arrangements (colour points) and their fit by the surface-selection rules with pseudo-refractive indices obtained from ellipsometry measurements (lines). Excitation wavelength was 532 nm. **Upper right:** Real and imaginary part of pseudo-refractive index $\tilde{n}=n+ik$ of silver NR arrays and their variation with both angles as measured by standard ellipsometry [112].

Another approach used for elucidation of polarization and angular dependences in SERS was employed in our recent work using MB adsorbed on silver NR arrays [112] (Figure 18). Although the nanostructures were morphologically anisotropic, the plasmonic properties around the excitation wavelength used (532 nm) were found rather isotropic. Therefore, it could not be the main reason for the anisotropic behaviour observed in the SERS experiments (anisotropy in the depolarization ratios measured under selected configurations). Instead, difference in the depolarization ratios after rotating the sample by 90° was attributed to different refractive indices along different directions (obtained using spectral ellipsometry) and explained within the framework of the surface selection rules. This fact was explained by slightly different periodicity along different directions instead of a specific angle made between the NR axes and incident/collected radiation. These results enabled quantitative analysis of MB Raman tensor elements, indicating that the molecules adsorb predominantly with the symmetry axis perpendicular to the surface. Interestingly, the depolarization ratio computed using Equation (24) produced the value of 0.29, which was in-between the depolarization ratio of MB molecules measured in a water solution (0.22) and the value of 1/3, predicted for a set of randomly-oriented hot-spots. It suggests that although the NRs were found plasmonically isotropic, the role of hot-spots is still manifested in the value of the depolarization ratio. The importance of the Fresnel mechanism-based enhancement at the metal-dielectric interface has also been highlighted in [126] where the origin of the additional SERS enhancement was explained on account of optical interference effects, giving rise to an enhancement ~ 3 times higher than in the case of direct illumination. The role of the surface-selection rules in SERS has also been experimentally demonstrated in [117] using a Nile blue molecule. By comparing the EF of various modes and relating these to their bare Raman tensors, the molecular orientation on a flat gold surface was inferred. Rich information about the Raman tensor components was obtained by SERS measurements as a function of the incident angle and both incident and scattered polarizations. The authors reported the EF up to ~ 3, but still retained the term SERS for simplicity. In this case, the EF was caused solely by the geometric factors and interpreted in terms of the Fresnel coefficients. This concept was extended in [127], investigating the wavelength and refractive index dependence of the geometrically enhanced SERS signal. In this approach, the effective optical constants of the nanostructured metallic film were determined by spectral ellipsometry measurements, applying a homogeneous uniaxial model. This approach highlights the need for application of the surface-selection rules not only in the case of smooth surfaces, but also in the case of plasmonic nanostructures. 

### 5.6. Plasmonic Anisotropy in Polarized Absorption and Emission and Their Influence on SERS

Anisotropic optical response of morphologically anisotropic plasmonic nanostructures is not only restricted to SERS. Polarization dependence of plasmon-enhanced fluorescence on individual Au NRs was reported in [99,128], producing maximum intensities with the excitation polarization oriented along the NR axis. Intensity profile obtained when the angle between the excitation polarization direction and the NR axis was varied was well fitted by a cosine squared function together with an exponential decay. Very similar trends were observed in [129] using Au NRs of different aspect ratios, or from a single Ag nanowire in [130]. Contradictory results appeared in [101]. 

Other instances where plasmonic anisotropy of metallic substrates is manifested include absorbance or reflectance measurements. Although the SERS properties of plasmonic nanostructures are very often derived from absorption or reflection measurements, this relationship works well only in the average SERS regime where the role of hot-spots is rather negligible, such as regular arrays of isotropic silver nanoislands [131]. In the case of more complicated (typically anisotropic and/or hot-spot dominated) plasmonic nanostructures, little correlation between these two phenomena (“near field” and “far field” properties of SERS substrates) was found [132,133]. Anisotropic SERS response surely is, in a more or less straightforward way, manifested in polarization-resolved absorbance or reflectance spectra with the difference in absorbance for different light polarization often even more pronounced than in the case of SERS. However, the exact relationship between the polarization providing the highest SERS enhancement and the polarization exhibiting the highest absorbance (or transmittance/reflectance) remains highly non-trivial. 

**Figure 19:**
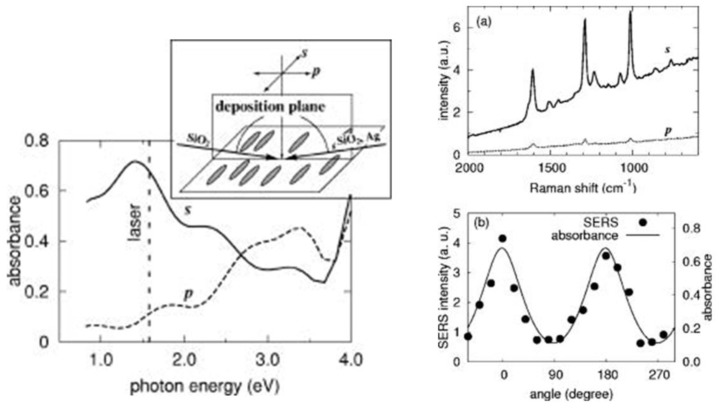
**Left:** The absorbance spectra of the Ag NR arrays tuned to the Raman spectroscopy at excitation wavelength of 785 nm (1.58 eV). The incident light was either p or s polarized, where an electric field vibrates parallel or perpendicular to the deposition plane, respectively, as shown schematically in the upper right box. **Right:** Polarization dependence of the Raman spectra (**a**) and the peak intensity at 1014 cm^−1^ (**b**). The polarization dependence of the absorbance at the wavelength of 785 nm is also shown (**b**). Adapted with permission from [98].

In the case of NR arrays, absorbance typically features a sharp transverse peak (for Ag generally below 400 nm) and a red-shifted broad longitudinal peak which spill one into another when rotating the polarization direction [97,112,134]. For aligned silver NRs in [98], the magnitude of the SERS enhancement and of absorbance of the same polarization were almost a perfect match (Figure 19) with the biggest response provided by polarization along the NRs. Counterintuitively, in the case of metallic grating structures [94], light polarization exhibiting the biggest transmission was also found to dominate in the SERS spectrum over perpendicular polarization for which the transmitted intensity was lower. Such trends were also found for silver NR arrays in [97], although a bigger SERS response was found for polarization perpendicular to the adjacent NRs. In a more elaborate study [135], the authors investigated changes in absorbance and its polarization characteristics as a function of the NR height, concluding that smaller NRs are rather isotropic. With the increase of the NR size (>1000 nm), absorbance splits into at least two plasmon resonant modes. 

The works mentioned above suggest that in the case of metallic NRs, the basic features or polarization-resolved absorbance is influenced by the specific preparation procedure of a given nanostructure less than polarization-resolved SERS. However, it has to be emphasized that polarization-resolved absorbance has been studied less intensively in literature than polarization-resolved SERS and many aspects regarding their mutual correspondence are yet to be fully resolved.

In a study devoted to angle resolved SERS on metallic nanostructured plasmonic crystals [136], a correlation between plasmon-related absorption in the reflectivity and the Raman enhancement with the varied angle of incidence was found. The need for absorbance or reflectance spectra may also be supplemented by ellipsometry measurements [112,116,127]. Although the plasmonic properties of silver NRs in [112] were found rather isotropic using the 532 nm wavelength, a clear distinction between the extinction profiles (computed using the ellipsometry characteristics) after rotating the sample by 90° was observed. This distinction was attributed to different periodicity in different directions as the NRs used were almost perfectly aligned in one direction, but still exhibiting slight deviations from the perfectly-ordered state in the perpendicular direction. This slight inhomogeneity was reflected in the inhomogeneously broadened longitudinal plasmon peak and was found responsible for orientation-dependent depolarization ratios of the adsorbed molecule, although the extinction profiles suggested rather plasmonic homogeneity. In [116], the SERS response for two different wavelengths was in agreement with the imaginary part of the effective dielectric function of the Ag film.

## 6. Conclusions

In this review, we have focused mainly on polarization and directional/angular characteristics occurring in Raman spectroscopy and surface-enhanced Raman spectroscopy (SERS) on anisotropic metallic nanostructures. We have summarized basic formulas for Raman intensities as a function of light polarization and angular arrangement used for both liquid samples as well as crystals. We have demonstrated that polarization- and angular-resolved measurements allow one to retrieve information on the (orientation-averaged) Raman tensor components, which are useful for symmetry determination and/or obtaining information regarding the molecular orientation. The complexity of this problem rises when molecules are adsorbed in the vicinity of nanostructured metallic surfaces, where not only coupling between light and molecules, but also (and often mainly) between light and the anisotropic NP arrays determine the total intensity profile. The observed spectral pattern results from interplay between the material used, size and shape of the nanoobjects, their spatial arrangement as well as the excitation wavelength used. Periodicity of the NP array is also deciding since it is manifested in the NP optical properties, which in turn influences the SERS signal. Since details of the NP fabrication are often very subtle, this process is very difficult to reproduce and therefore the exact results often vary in literature. It is one of the reasons why all aspects of polarization- and angular resolved measurements are not yet fully understood and remain a challenge for future research. It is expected that study of the polarization and angular properties of molecules on plasmonic nanostructures will contribute to a deeper theoretical understanding of the enhancement mechanism. It will also provide a theoretical background for the design of plasmon-based molecular sensors for maximum signal enhancement.
